# Supplementary material for: Prospective observational study of SARS-CoV-2 infection, transmission and immunity in a cohort of households in Liverpool City Region, UK (COVID-LIV): a study protocol
Source: BMJ Open. 2021 Mar 17;11(3):e048317. doi: 10.1136/bmjopen-2020-048317 (PMC7977072; doi:10.1136/bmjopen-2020-048317)
Supplement: Supplementary data [file bmjopen-2020-048317supp001.pdf]

COVID-LIV Study

UNIVERSITY OF LIVERPOOL

Liverpool Household COVID-19 Cohort Study (COVID-LIV)

Adult Information Sheet for COVID-LIV

- You were contacted by telephone or you have contacted us and verbally agreed to take part in a research study called COVID-LIV. You also provided information about yourself.
- Please now take time to read the following information carefully (note, you may have received this information via email following the telephone call). **Part 1** tells you the purpose of the study and what will happen to you if you take part. **Part 2** gives you more detailed information about the conduct of the study.
- You can ask a member of the research team if there is anything that is not clear, or if you would like more information.
- Taking part is voluntary. If you don't want to take part then you don't need to give a reason.
- If you want to take part but other members of the household do not want to, we may not be able to include you in the study.
- The COVID-LIV study team are studying how COVID-19 spreads in the community, inside households.
- This is being done using swab tests – the same tests that you would have if you came into hospital with COVID-19. If you choose to, you can also provide a stool sample at 6-8 and 12-14 weeks.
- We need all kinds of different households across Liverpool city region to take part.
- The study is currently funded for 12 months whilst additional funding is obtained to continue for a further three years.

How to contact the study team:

If you have any questions about this study, please talk to a member of the study team who visits you, or call: ###

Professor Neil French is the lead Investigator.

Contents:

Part 1 – Purpose of the study and what will happen if you take part

- **Why are we doing the COVID-LIV study?**
- **Why have I been chosen?**
- **Do I have to take part?**
- **What will happen to me if I take part?**
- **What will I have to do if I take part?**
- **What are the benefits and risks of taking part?**
- **What happens if I change my mind?**
- **What happens when the study stops?**
- **What if there is a problem?**
- **Will my taking part in the study be kept confidential?**

Part 2 – Detailed information about the conduct of the study

- **Who is running the study?**
- **How will my information be collected and handled?**
- **What are my choices about how my information is used?**
- **Information sharing for other research**
- **Where can I find out more about how my information is used?**
- **What will happen to the nose and throat swabs, and the blood, saliva and stool samples I give?**
- **What if there is a problem?**

If you become ill or suspect you have COVID-19 please follow government guidelines

Setiabudi W, *et al. BMJ Open* 2021; 11:e048317. doi: 10.1136/bmjopen-2020-048317

PART 1: Purpose of the study and what will happen if you take part

Why are we doing the COVID-LIV study?

COVID-19 (or COVID for short) is the name of the disease that is caused by the new coronavirus. This coronavirus was first found in China in January 2020. The new coronavirus has since spread all round the world. COVID-19 is caused by a virus strain called SARS-CoV-2. We want to understand what factors determine transmission of the virus and how our bodies respond and become resistant to it.

Most people who get COVID-19 will be fine as it is not a serious disease in most people. Some people might not even know that they had it. But in a few people, COVID-19 makes them very sick. They may need a ventilator to help them breathe, or may even die. In order to prevent this, the government has asked everyone except essential workers to stay at home whenever possible. Whilst this will work, it has other effects, for example preventing people from working, so there is a great cost to the country, and to our personal freedoms. We need to understand how COVID-19 spreads, so we can help tell the government when to stop advising people to stay home, and what might happen as they do tell us we can go out again.

The results from this study will be used to provide valuable information for the government and local public health to plan the next stage of the COVID-19 response – that is how we step down from the lock down and back to more contacts and interactions.

Why have I been chosen?

You, and your household, have either been selected because you were part of another study before, called CLAHRC NWC Household Health Survey or you have responded to one of our communications seeking volunteers. Those in the household study gave permission to be re-contacted again about other studies. Therefore, we are contacting you to ask if you would like to take part in this study.

We are selecting different types of households (for example with different numbers of people, or those with and without children) to take part in COVID-LIV. We need all different kinds to take part so we have not approached you based on anything particular about you or your household, or family. We are looking for around 1000 people from 300 different households to take part, so that we can be sure the people in the study are just like those in the whole community.

Do I have to take part?

No, taking part is voluntary. It is up to you to decide whether or not you want to take part.

We also want all the members of the household to take part. If you want to but other members of the household do not want to participate we may not be able to include you.

If you do decide to take part, we will ask you to sign a consent form.

If you decide to take part, you can also choose to stop at any time without giving a reason.

The decision you make on whether to take part or not will not affect the care you receive for COVID-19 now, or in the future.

What will happen to me if I take part?

After you have signed the consent form, we will ask you to complete a questionnaire (we will ask you about members of your household, diet, employment, and some other things). The researcher will take up to 60ml blood sample from you. This sounds like a lot, but it is in fact only about 1½ egg cups in size. For the participants unable to donate blood, the researcher will take a finger prick test and blood spot collection. We will also collect a sample of saliva with a mini sponge and a sample of nasal fluid with a small piece of special filter paper. We will then explain how you can take nose swabs from yourself or other members of the household and ask you to do the first swab in the presence of a researcher to check you are able to do it correctly. These swabs are for COVID-19 testing, and are easy to use. If you feel ill at the time of the first visit we will only take the nose swab to see if you have COVID19 and arrange a further visit to collect blood and other samples depending on the swab result. You will swab your nose once a week, for 12 weeks (or at any other time if you think you have fallen

Setiabudi W, *et al. BMJ Open* 2021; 11:e048317. doi: 10.1136/bmjopen-2020-048317

ill with fever, cough, sore throat, breathlessness or flu-like symptoms). We will phone you, or send a text message, to remind you to take the nose swabs once a week. If you struggle to take the nose swabs yourself, simply let us know and we can arrange for the researcher to visit you and help take the swabs.

We will let you know when a courier will collect the swabs from you – this will happen on a weekly basis. The swabs will be taken to a laboratory team who will do COVID-19 tests on them. This takes up to three days, and we will phone you, or send a text message, with the results.

After the first 12 weeks, we do not need swabs every week. However, we would still like you to collect nose swabs at any point in the study if you think you have fallen ill with fever, cough, sore throat, or breathlessness. At this point, the processing of swabs may change depending on local NHS testing policy

The researcher will visit and collect up to 60ml blood samples after you have been in the study for 12 weeks (3 months), then again at 6 months, 1 year, 2 years and 3 years after you started the study. For the participants unable to donate blood, the researcher will take a finger prick test and blood spot collection. At these visits, you will also complete a questionnaire.

If a nose swab has a positive test result: *We will provide support in terms of your healthcare (either via the phone, or face-to-face), and we will also visit you a few days after your positive result, and then 2 weeks after that. We will inform your GP of your positive result. Public Health England maybe informed in line with their current guidelines. When we visit, we will ask you more questions, and take some more samples, both from the person in the household who has tested positive, and from the rest of the household as well. The samples we will take are: another nose swab, a swab for saliva, a stool sample, and further blood samples (up to 60ml). We may also provide you with a special device for rapid diagnosis to take a nose/throat swab, but this is only if it is approved for use and part of NHS guidelines. If another member of the household tests positive, then we will take additional blood, saliva, nose and throat samples off them as well. The maximum number of times we take samples would be about 4 in a month.*

If, at any point, you become ill or suspect you have COVID-19, you can call the telephone number given on the first page of this information sheet. If you suspect you have COVID-19 and need to talk to someone outside of usual office hours, you should call **NHS ###.**

The study is planned to last for three years. At the moment the study is going to be started for the first 12 weeks (3 months); continuing the study after that will depend on the study team obtaining more funding. However, our intention is to run the study for three years, so we are asking for permission for this up front from you, so that we do not need to keep coming back to you.

We will let you know when the study ends.

What will I have to do if I take part?

If you agree to take part, you will be asked to sign a consent form. You will be provided with a copy of the consent form and the information sheet to keep.

Once you have signed the consent form, you will be asked to follow the study plan (see study timeline diagram below).

You will have to:

- Provide swab of the throat and nose at the beginning, repeated weekly for the first 12 weeks and at any time point you have fallen ill with fever, cough, sore throat, or breathlessness taken by yourself
- Be ready at pre-set timepoints for the courier to collect the swabs (once a week)
- Provide blood samples (or finger prick test, if unable to donate blood)
- Provide saliva swabs (only if you test positive for COVID-19)
- Provide stool sample at week 6-8 and 12-14 (optional), and if you test positive for COVID-19)
- Complete questionnaires (either by yourself or over the telephone)
- Provide information on your health and wellbeing

Setiabudi W, *et al. BMJ Open* 2021; 11:e048317. doi: 10.1136/bmjopen-2020-048317

Initial contact

Joining COVID-LIV

- Potential participants are contacted for the study team to give a brief explanation about the study, answer any queries and acquire verbal consent.
- A visit by a team of research nurse or doctor is arranged.

Week 0

Initial visit

- The study team will answer any questions and acquire written informed consent.
- Detailed questionnaire will be filled
- Participants will provide blood sample (or fingerprick), nose and throat swabs, nasal strip and saliva.
- The study team will explain how to take self-administered nose swabs and provide with swab kits for the next weeks

Weeks 1 - 11

Weekly questionnaires and swabs

- Participant will fill a brief questionnaire
- Participants will take weekly nose swab
- Participant will provide an optional stool samples at week 6-8.
- Courier will come and collect the samples every week.

Week 12

Questionnaire, blood sample and swabs

- You will complete a detailed questionnaire.
- If you choose to, you can also provide a stool sample at week 12-14.
- We will take a blood sample (or fingerprick), and a nose swab.
- We will phone/text you with the results in 2/3 days.

If you test positive (study nurse visits you)

If you test positive for COVID-19 (this visit will occur a few days later, and again about 2 weeks after that)

We will inform your GP of the positive result.
We will ask some questions about your health.
We will take a blood sample, nose swab, throat swab, nasal strip, a swab for saliva and a stool sample from all members of the household where someone has tested positive. If another person tests positive more samples will be taken off the household, up to 4 in a month.
Also at 3, 6, 12, 24 and 36 months.
We may also take a nose/throat swab using a special device, but this is only if it is approved for us to use.

If additional funding is received, and the study continues

If the study continues past 3 months, this visit will occur 6 months, 1 year, 2 years and 3 years after you consented to join the study

You will complete a detailed questionnaire.
We will take a blood sample.

12 weeks, or up to 3 years, after you join the study

End of your participation

No further visits/samples/data collection will occur once you have reached this point, unless additional funding is received.

Setiabudi W, *et al. BMJ Open* 2021; 11:e048317. doi: 10.1136/bmjopen-2020-048317

What are the benefits and risks of taking part?

The main benefit is that you will know if you have had COVID-19 or not. If you have symptoms, and the test is negative, we will tell you this. This means that, once you have **self-isolated for 7 days** (as per government advice at the beginning of June, if this changes we will let you know), you could still go out to work or to the shops because we will know for sure that you have not got COVID-19. We will also be doing antibody tests on your blood, so you will be tested for immunity as well. However, at the moment we are not sure if these tests means that you will actually be protected from repeat infections in the future. Studying that is one of the aims of the study.

The risks are minor bruising from the blood samples taken, and researchers entering your house. However, the researchers will be wearing Personal Protective Equipment (PPE) at all times, to prevent transfer of COVID-19.

What happens if I change my mind?

If at any point you decide to stop taking part in the study, you can let a member of the research team know. In order for us to understand why participants withdraw from the study, we may ask you why you have decided to withdraw. However, you do not have to give a reason, if do not want to.

If you do decide to stop taking part, this will not affect your current and future medical care, and your legal rights will not be affected in any way.

Information on how we will handle your information in the event of you withdrawing is detailed in Part 2 of this Information Sheet.

What happens when the study stops?

It is intended that the results of the study will be presented at conferences and published in medical journals so that we can explain to the medical community what our research results have shown. Confidentiality will be ensured at all times and you will not be identified in any publication.

Any information derived directly or indirectly from this research, as well as any patents, diagnostic tests, drugs, or biological products developed directly or indirectly as a result of this research may be used for commercial purposes. You have no right to this property or to any share of the profits that may be earned directly or indirectly as a result of this research. However, in signing this form and donating swabs and blood samples for this research, you do not give up any rights that you would otherwise have as a participant in research.

What if there is a problem?

If, at any point, you become ill or suspect you have COVID-19, you can call the telephone number given on the first page of this information sheet. If you suspect you have COVID-19 and need to talk to someone outside of usual office hours, you should call **NHS ###**.

Any complaint about the way you have been dealt with during the study or any possible harm you might suffer will be addressed. Detailed information is given in Part 2 of this information sheet.

Will my taking part in the study be kept confidential?

Yes. All the confidential information about your participation in this study will be kept confidential. Detailed information on this is given in Part 2.

Setiabudi W, *et al. BMJ Open* 2021; 11:e048317. doi: 10.1136/bmjopen-2020-048317

PART 2: Detailed information about the conduct of the study

Who is running the study?

University of Liverpool is the Sponsor of this study and is responsible for managing it. They are based in United Kingdom. They have asked that the day to day running of the study is carried out by a team based at the Liverpool Clinical Trials Centre (LCTC, part of the University of Liverpool), and the samples you provide will be managed by members of a laboratory team at the University of Liverpool and Liverpool School of Tropical Medicine (LSTM).

The study has been reviewed by the National Research Ethics Service Committee to make sure that the study is scientifically and ethically acceptable.

This study is funded by the National Institute for Health Research (NIHR) Health Protection Research Unit in Emerging and Zoonotic Infections (HPRU-EZI), Centre of Excellence in Infectious Diseases Research (CEIDR) at University of Liverpool in partnership with Public Health England (PHE), Alder Hey Charity and in collaboration with Liverpool School of Tropical Medicine The views expressed are those of the author(s) and not necessarily those of the NIHR or the Department of Health and Social Care.

We will not receive any payment for including you in this study.

How will my information be collected and handled?

University of Liverpool is the Data Controller for this study and will need to use information from this research project.

This information will include your name, initials, date of birth, contact details, postcode and your NHS number (we will request this from your GP). People will use this information to do the research or to check your records to make sure that the research is being done properly.

Individuals from University of Liverpool, the LCTC, LSTM, and relevant regulatory organisations may look at your research records to check the accuracy of the research study.

People who do not need to know who you are will not be able to see your name or contact details. Your data will have a code number instead.

Data will be sent from the University of Liverpool researchers and LSTM to the LCTC. Data may also be sent from your GP to University of Liverpool researcher, who will then send this to LCTC.

We may notify your GP that you are taking part in the study, and if you test positive for COVID-19, for their information.

We will keep all information about you safe and secure.

Once we have finished the study, we will keep the data for a minimum of 10 years, so we can check the results. We will write our reports in a way that no-one can work out that you took part in the study.

What are my choices about how my information is used?

You can stop being part of the study at any time, without giving a reason, but we will keep information about you that we already have.

We need to manage your records in specific ways for the research to be reliable. This means that we won't be able to let you see or change the data we hold about you.

Information sharing for other research

When you agree to take part in a research study, the information about your health and care may be beneficial to researchers running other research studies in this organisation and in other organisations. These organisations may be universities, NHS organisations or companies involved in health and care research in this country or abroad. Your information will only be used by organisations and researchers to conduct research in accordance with the UK Policy Framework for Health and Social Care Research, or equivalent standards.

If you agree to take part in this study, you will have the option to take part in future research using your data saved from this study.

Setiabudi W, *et al. BMJ Open* 2021; 11:e048317. doi: 10.1136/bmjopen-2020-048317

Where can I find out more about how my information is used?

You can find out more about how we use your information:

- at the LCTC website: www.lctc.org.uk/privacy
- at LSTM website: https://www.lstmed.ac.uk/lstm-privacy-statement
- at www.hra.nhs.uk/information-about-patients
- in the Health Research Authority leaflet available from www.hra.nhs.uk/patientdataandresearch
- by contacting the University of Liverpool Data Protection Officer on LegalServices@liverpool.ac.uk
- by asking one of the research team
- by sending an email to COVID-LIV-FWCom@liverpool.ac.uk, or
- by ringing us on ###

What will happen to the nose and throat swabs, and the blood, saliva and stool samples I give?

Samples collected for use in COVID-LIV

We will use your samples to test for your body's response to the COVID-19 virus. This will be able to tell us whether you have had the infection with no symptoms, in some cases. We will test your blood to see if you have been exposed before to other coronaviruses.

Your samples will be sent to laboratories at the University of Liverpool and LSTM for analysis. These samples will be coded and the researchers carrying out tests on the samples will not be given information they do not need to carry out the tests and analyse the results. Coded is not the same as anonymous. The study ream will need to know who the sample came from to inform you of the results. It will be possible to use the codes to identify that a result is from your sample. However, once we have given you your results, we do not plan to do this unless there is a good reason to do so. We will maintain this information so that we can properly manage the samples. For instance, sometimes we may need to update our record of your clinical details to help us interpret the results of tests.

If you or a member of your household tested positive for COVID-19: We will use your samples for much more detailed and complex tests on the body's response to the COVID-19 virus. We will study the body's response for everyone in the household – this will help understand how people become infected and how some people may resist the virus. We may also test your blood with other viruses as controls, such as the glandular fever virus, flu, and some other common cold viruses. We will look in detail at the responses of specialised white blood cells in your blood called lymphocytes.

We may store some of your blood, or the cells from your blood for up to 25 years after this research has finished. We may also use some of your cells to make what are called "cell lines" – these are cells that can be kept alive in the lab for a long time (maybe forever) and are used to make it easier to detect and study the lymphocyte responses we are interested in.

We will extract DNA to look at your genes by sequencing the whole of your genome. This will help us understand whether certain genes are related to your ability to fight off the COVID-19 coronavirus. This will include some of the genes unique to your individual immune system, called HLA. These are the same tests that are done before organ transplants, and they are used to tailor our research to each person. There will be left over DNA after we have done this, which would be stored, like the cells from your blood, for up to 25 years after the research has finished.

Samples collected for Future Research

We would like your permission to do other research on these stored samples in the future. This would include looking at factors which are involved in fighting off coronaviruses, and other controls for our experiments such as herpes viruses (like glandular fever), flu, and enteroviruses (e.g. common cold viruses), and other common human viruses. We cannot say now all the experiments we might do because new things might be discovered in the future that we would like to investigate.

If you agree that we can store your samples for future research, coded samples will be stored at the University of Liverpool and LSTM. These researchers work closely with other scientists in the UK and elsewhere. We would like to allow other researchers, including those

Setiabudi W, *et al. BMJ Open* 2021; 11:e048317. doi: 10.1136/bmjopen-2020-048317

who also have ideas about coronaviruses, to apply to use your samples for similar work in the future too.

We would like your permission to allow your samples to be transferred outside of the University of Liverpool and LSTM for purposes including those of coronavirus immunity testing. We are asking this now so we don't have to ask you again in the future.

Any future experiments not related to coronaviruses or other common human virus infections would be approved by a research ethics committee. If you don't want your samples stored, that's fine, you can still take part in the rest of the study, we just won't keep your samples at the end. Or if you are happy with having your samples stored, but not sent to another lab, you can choose this as well.

The samples will be kept in a secure place until we need them; nobody outside of the study will have access to **any** confidential information that you give to us. Confidential details (such as your name, address and GP details) will be kept locally and not made available to collaborators.

What if there is a problem?

If you have a concern about any aspect of this study, you should ask to speak with one of the research team who will do their best to answer your questions.

Every care will be taken in the course of this research study. However, in the unlikely event that you are harmed by taking part in this research project of the study Sponsor (University of Liverpool), the Sponsor holds Insurance for the conduct of clinical research. Compensation may be available and you may have to pay your related legal costs.

Thank you for taking the time to read and consider this information sheet. Should you decide to take part in the study, you will be given a copy of the information sheet and a signed consent form to keep.

Setiabudi W, *et al. BMJ Open* 2021; 11:e048317. doi: 10.1136/bmjopen-2020-048317

COVID-LIV Study

UNIVERSITY OF LIVERPOOL

Liverpool Household COVID-19 Cohort Study (COVID-LIV)

FOR SITE USE ONLY:

Site Name:			
Household ID:		Participant ID	
Participant Initials:		Participant DOB:	/ /
Participant Postcode:	-		

Adult Consent Form

To be completed by the participant:

Once you have read and understood each statement please enter your initials in each box.	Initial
1. I have read and understood the information sheet for this study. I have had the opportunity to ask questions and have had these answered satisfactorily.	
2. I understand that participation is voluntary and that I am free to withdraw from the study at any time, without giving a reason, and without my care or legal rights being affected. However, the study team may need to collect some limited information for safety reasons.	
3. I give permission for a copy of this fully completed consent form to be sent to the LCTC (where it will be kept in a secure location) to allow confirmation that my consent was given.	
4. I understand that relevant sections of my medical notes and any data collected during the study may be looked at by authorised individuals from the central study team and representatives of the Sponsor, and relevant regulatory authorities. I give permission for these individuals to have access to my records and data.	
5. I understand that my data will be kept by the University of Liverpool and all others archiving data in a confidential manner for up to a maximum of 25 years from the end of the study.	
6. I consent to samples of my blood, saliva and stool, and for nose and throat swabs to be taken and used for this study.	
7. I agree to take part in the above study.	
The statements below are optional (you can still take part in the study even if you do not wish to agree to these):	
8. I agree for data previously collected for other research to be looked at by the research team.	
9. I consent to providing an optional stool sample at week 6-8 and 12-14 for the purpose of this study.	
10. I consent to my blood and stool samples being stored by the University of Liverpool for testing for SARS-CoV-2 immunity, and immunity to other coronaviruses.	
11. I agree for samples collected for future research to be stored at The University of Liverpool and LSTM.	
12. I agree for samples collected for future research to be transferred outside of the University of Liverpool and LSTM, along with a copy of this Consent Form, for different tests for coronavirus immunity.	
13. I agree to allow information or results arising from this study to be used in future healthcare and/or medical research providing my confidentiality is maintained.	
14. I agree to my GP being informed of my participation in the study, and if I test positive for COVID-19.	
15. I agree for my GP to be contacted for information relating to my health records, including the provision of my NHS number.	

Setiabudi W, *et al. BMJ Open* 2021; 11:e048317. doi: 10.1136/bmjopen-2020-048317

COVID-LIV Study

UNIVERSITY OF LIVERPOOL

Liverpool Household COVID-19 Cohort Study (COVID-LIV)

FOR SITE USE ONLY:

Site Name:			
Household ID:		Participant ID	
Participant Initials:		Participant DOB:	/ /
Participant Postcode:	-		

Adult Consent Form

16. I agree that I may be contacted in the future in relation to this or other related studies. ☐
If you agree to statement 15 provide your details below:

Telephone number:	
Email address:	

To be completed by the participant:

Your full name (please print):			
Your signature:		Date:	

To be completed by the Researcher (after participant has completed the form):

Researcher full name (please print):			
Researcher signature:		Date:	

Please file the original wet-ink copy in the COVID-LIV Investigator Site File, and make two copies: one for the participant and one to be sent to the LCTC.

Setiabudi W, *et al. BMJ Open* 2021; 11:e048317. doi: 10.1136/bmjopen-2020-048317
