# Supplementary material for: Prospective observational study of SARS-CoV-2 infection, transmission and immunity in a cohort of households in Liverpool City Region, UK (COVID-LIV): a study protocol
Source: BMJ Open. 2021 Mar 17;11(3):e048317. doi: 10.1136/bmjopen-2020-048317 (PMC7977072; doi:10.1136/bmjopen-2020-048317)
Supplement: Author's manuscript [file bmjopen-2020-048317.draft_revisions.pdf]

BMJ Open

BMJ Open is committed to open peer review. As part of this commitment we make the peer review history of every article we publish publicly available.

When an article is published we post the peer reviewers' comments and the authors' responses online. We also post the versions of the paper that were used during peer review. These are the versions that the peer review comments apply to.

The versions of the paper that follow are the versions that were submitted during the peer review process. They are not the versions of record or the final published versions. They should not be cited or distributed as the published version of this manuscript.

BMJ Open is an open access journal and the full, final, typeset and author-corrected version of record of the manuscript is available on our site with no access controls, subscription charges or pay-per-view fees (http://bmjopen.bmj.com).

If you have any questions on BMJ Open's open peer review process please email info.bmjopen@bmj.com

BMJ Open

Prospective observational study of SARS-CoV-2 infection, transmission, and immunity in a cohort of households in Liverpool City Region, UK (COVID-LIV): a study protocol

Journal:	*BMJ Open*
Manuscript ID	bmjopen-2020-048317
Article Type:	Protocol
Date Submitted by the Author:	22-Dec-2020

Complete List of Authors:	Setiabudi, Wega; University of Liverpool, Department of Clinical Infection, Microbiology and Immunology, Institute of Infection, Veterinary and Ecological Sciences<br>Hungerford, Daniel; University of Liverpool, Department of Clinical Infection, Microbiology and Immunology, Institute of Infection, Veterinary and Ecological Sciences; NIHR HPRU EZI<br>Subramaniam, Krishanthi; University of Liverpool, Department of Clinical Infection, Microbiology and Immunology, Institute of Infection, Veterinary and Ecological Sciences<br>Vaselli, Marcella; University of Liverpool, Department of Clinical Infection, Microbiology and Immunology, Institute of Infection, Veterinary and Ecological Sciences<br>Shaw, Victoria E.; University of Liverpool, Good Clinical Laboratory Practice, Institute of Translational Medicine<br>Wilton, Moon; University of Liverpool, Department of Psychology, Institute of Population Health<br>Vivancos, Roberto; Public Health England, Field Epidemiology North West, Field Service, National Infection Service; NIHR HPRU EZI<br>Aston, Stephen; University of Liverpool, Department of Clinical Infection, Microbiology and Immunology, Institute of Infection, Veterinary and Ecological Sciences<br>Platt, Gareth; University of Liverpool, Department of Clinical Infection, Microbiology and Immunology, Institute of Infection, Veterinary and Ecological Sciences<br>Moitt, Tracy; University of Liverpool, Liverpool Clinical Trial Centre<br>Jones, Ashley P.; University of Liverpool, Liverpool Clinical Trial Centre<br>Gabbay, Mark; University of Liverpool, Department of Primary Care and Mental Health, Institute of Population Health; NIHR HPRU EZI<br>Buchan, Iain; University of Liverpool, Department of Public Health and Policy, Institute of Population Health<br>Carrol, Enitan; University of Liverpool, Department of Clinical Infection, Microbiology and Immunology, Institute of Infection, Veterinary and Ecological Sciences<br>Iturriza-Gomara, Miren; University of Liverpool, Department of Clinical Infection, Microbiology and Immunology, Institute of Infection, Veterinary and Ecological Sciences<br>Solomon, Tom; University of Liverpool, Department of Clinical Infection, Microbiology and Immunology, Institute of Infection, Veterinary and Ecological Sciences; NIHR HPRU EZI

Greenhalf, William; University of Liverpool, Liverpool Good Clinical Laboratory Practice, Institute of Translational Medicine
Naisbitt, Dean J.; University of Liverpool, Department of Molecular and Clinical Pharmacology, Institute of Translational Medicine
Adams, Emily ; Liverpool School of Tropical Medicine, Department of Tropical Disease Biology; NIHR HPRU EZI
Cunliffe, Nigel; University of Liverpool, Department of Clinical Infection, Microbiology and Immunology, Institute of Infection, Veterinary and Ecological Sciences
Turtle, Lance; University of Liverpool, Department of Clinical Infection, Microbiology and Immunology, Institute of Infection, Veterinary and Ecological Sciences; NIHR HPRU EZI
French, Neil; University of Liverpool, Department of Clinical Infection, Microbiology and Immunology, Institute of Infection, Veterinary and Ecological Sciences; NIHR HPRU EZI

Keywords: COVID-19, IMMUNOLOGY, Epidemiology < INFECTIOUS DISEASES, Respiratory infections < THORACIC MEDICINE, VIROLOGY

SCHOLARONE™
Manuscripts

I, the Submitting Author has the right to grant and does grant on behalf of all authors of the Work (as defined in the below author licence), an exclusive licence and/or a non-exclusive licence for contributions from authors who are: i) UK Crown employees; ii) where BMJ has agreed a CC-BY licence shall apply, and/or iii) in accordance with the terms applicable for US Federal Government officers or employees acting as part of their official duties; on a worldwide, perpetual, irrevocable, royalty-free basis to BMJ Publishing Group Ltd ("BMJ") its licensees and where the relevant Journal is co-owned by BMJ to the co-owners of the Journal, to publish the Work in this journal and any other BMJ products and to exploit all rights, as set out in our licence.

The Submitting Author accepts and understands that any supply made under these terms is made by BMJ to the Submitting Author unless you are acting as an employee on behalf of your employer or a postgraduate student of an affiliated institution which is paying any applicable article publishing charge ("APC") for Open Access articles. Where the Submitting Author wishes to make the Work available on an Open Access basis (and intends to pay the relevant APC), the terms of reuse of such Open Access shall be governed by a Creative Commons licence – details of these licences and which Creative Commons licence will apply to this Work are set out in our licence referred to above.

Other than as permitted in any relevant BMJ Author's Self Archiving Policies, I confirm this Work has not been accepted for publication elsewhere, is not being considered for publication elsewhere and does not duplicate material already published. I confirm all authors consent to publication of this Work and authorise the granting of this licence.

For peer review only

TITLE

Prospective observational study of SARS-CoV-2 infection, transmission, and immunity in a cohort of households in Liverpool City Region, UK (COVID-LIV): a study protocol

AUTHORS

Wega Setiabudi[1]*, Daniel Hungerford[1,2,3]*, Krishanthi Subramaniam[1], Marcella Vaselli[1], Victoria E. Shaw[4], Moon Wilton[5], Roberto Vivancos[2,3,6], Stephen Aston[1], Gareth Platt[1], Tracy Moitt[7], Ashley Jones[7], Mark Gabbay[3,8], Iain Buchan[2,9], Enitan D. Carrol[1], Miren Iturriza-Gomara[1,2], Tom Solomon[1,3], William Greenhalf[4], Dean J. Naisbitt[10], Emily R. Adams[3,11], Nigel A. Cunliffe[1,2], Lance Turtle[1,3], Neil French[1,3], on behalf of the COVID-LIV Study Group

Affiliations

1. Department of Clinical Infection, Microbiology, and Immunology, Institute of Infection, Veterinary, & Ecological Sciences, University of Liverpool, Liverpool, UK
2. NIHR HPRU in Gastrointestinal Infections at the University of Liverpool, Liverpool, UK
3. NIHR HPRU in Emerging and Zoonotic Infections at the University of Liverpool, Liverpool, UK
4. Liverpool Good Clinical Laboratory Practice, Institute of Translational Medicine, University of Liverpool, Liverpool, UK
5. Department of Psychology, Institute of Population Health, University of Liverpool, Liverpool, UK
6. Field Epidemiology North West, Field Service, National Infection Service, Public Health England, Liverpool, UK
7. Liverpool Clinical Trial Centre, University of Liverpool, Liverpool, UK
8. Department of Primary Care and Mental Health, Institute of Population Health, University of Liverpool, Liverpool, UK
9. Department of Public Health and Policy, Institute of Population Health, University of Liverpool, Liverpool, UK
10. Department of Molecular and Clinical Pharmacology, Institute of Translational Medicine, University of Liverpool, Liverpool, UK
11. Department of Tropical Disease Biology, Liverpool School of Tropical Medicine, Liverpool, UK

Corresponding Author:
Neil French. 8 West Derby Street, Liverpool, L69 7BE, United Kingdom. french@liverpool.ac.uk

*WS and DH contributed equally to this paper.

*WS and DH are joint first authors.

For peer review only

ABSTRACT

Introduction

The emergence and rapid spread of COVID-19 have caused widespread and catastrophic public health and economic impact, requiring governments to restrict societal activity to reduce the spread of the disease. The role of household transmission in the population spread of SARS-CoV-2, and of host immunity in limiting transmission, is poorly understood. This paper describes a protocol for a prospective observational study of a cohort of households in Liverpool City Region, UK, which addresses the transmission of SARS-CoV-2 between household members and how immunological response to the infection changes over time.

Methods and analysis

Households in the Liverpool City Region, in which members have not previously tested positive for SARS-CoV-2 with a nucleic acid amplification test, are followed up for an initial period of 12 weeks. Participants are asked to provide weekly self-throat and nasal swabs and record their activity and presence of symptoms. Incidence of infection and household secondary attack rates of COVID-19 are measured. Transmission of SARS-CoV-2 will be investigated against a range of demographic and behavioural variables. Blood and faecal samples are collected at several time points to evaluate immune responses to SARS-CoV-2 infection and prevalence and risk factors for faecal shedding of SARS-CoV-2, respectively.

Ethics and dissemination

The study has received approval from the NHS Research Ethics Committee; REC Reference: 20/HRA/2297, IRAS Number: 283464. Results will be disseminated through scientific conferences and peer-reviewed open access publications. A report of the findings will also be shared with participants. The study will quantify the scale and determinants of household transmission of SARS-CoV-2. Additionally, immunological responses before and during the different stages of infection will be analysed, adding to the understanding of the range of immunological response by infection severity.

STRENGTHS AND LIMITATIONS OF THIS STUDY

- COVID-LIV is a prospective cohort study of households that aims to represent the socioeconomic profile of Liverpool City Region population, which enables the determination of risk factors of SARS-CoV-2 transmission whilst minimising recall bias.
- This household-based study will identify paucisymptomatic and asymptomatic COVID-19 cases, thus allowing the measurement of their contribution to transmission.
- The longitudinal nature of the study enables the capture of subjects before they test positive for COVID-19, which provides a pre- and post-infection time point to evaluate changes to the host immune response.
- Limitations include the relatively small sample size and repeated self-sampling, which may lead to diagnostic inconsistencies.
- Participation bias by those most engaged with COVID-19 and disease control may theoretically result in an unrepresentative study cohort.

For peer review only

INTRODUCTION

Within months of the first reports of a novel respiratory disease in Wuhan, China in December 2019, COVID-19 has been declared a global pandemic with devastating impacts.[1] The disease caused by the severe acute respiratory syndrome coronavirus 2 (SARS-CoV-2) has reached 46.8 million cases and 1.2 million deaths globally as of 3 November 2020, although the real number is likely to be much higher.[2] With limited evidence of effective prophylactic treatment and prior to availability of vaccination, countries around the world have been forced to implement various forms of restriction to individual movement in order to control the transmission of SARS-CoV-2.[3–5] Although the effectiveness of such measures in controlling viral transmission comes at the cost of disruption in socioeconomic activity and mental wellbeing, the impact from uncontrolled transmission could cause the loss of millions of lives and the potential collapse of health systems.[6–9]

Understanding the pattern of community transmission is essential to inform approaches to contain the spread of SARS-CoV-2. The role of transmission between household members is believed to have a significant role in the spread of the disease, where the secondary attack rate is estimated to be 16.6%.[10] This is reflected in the current UK government guideline where members of the household of a confirmed case are required to self-isolate for ten days.[11] Despite this, the limited availability of long-term prospective cohort studies means that further exploration of how SARS-CoV-2 transmits within households, and a better understanding of how immune responses develop over time, are urgently needed.[10,12]

In October 2020, the Liverpool City Region became the first area in England, UK to be placed in the highest level of regional restriction after experiencing one of the highest rate of infection in the country.[13] The region was also chosen as the site for the pilot asymptomatic mass testing due to its high infection rate during the second national lockdown.[14] The transmission characteristic of the Liverpool City Region could be explored further through a community study of the virus transmission. These data would aid understanding of the transmission dynamics of SARS-CoV-2 that may be beneficial in informing public health interventions.

The Liverpool Household COVID-19 Cohort Study (COVID-LIV) is a prospective observational study of households in the Liverpool City Region. As a household-based study, COVID-LIV will capture paucisymptomatic and asymptomatic COVID-19 cases. This allows measurement of the role of different disease manifestation of COVID-19 cases in the transmission of SARS-CoV-2 between household members. In addition, the prospective nature of the study allows characterisation of the immune response to SARS-CoV-2 at different stages of the infection and determine the durability of the response.

STUDY AIMS

Among households in the Liverpool City Region, the primary aim is to understand household associated SARS-CoV-2 transmission. This aim will be achieved by:

1. Measurement of household COVID-19 incidence and secondary attack rates
2. Identification of the determinants of transmission of SARS-CoV-2
3. Estimation of the contribution of paucisymptomatic and asymptomatic infection to the spread of SARS-CoV-2

For peer review only

Secondary aims are:

1. Measure family member contact patterns and the relationship to household structure
2. Describe the clinical phenotype of mild COVID-19 cases
3. Undertake sequence SARS-CoV-2
4. Characterise the immune response in mild COVID-19 cases
5. Characterise the immune response of exposed household contacts with no subsequent detection of confirmed infection
6. Investigate the prevalence of household faecal shedding of SARS-CoV-2

METHODS AND ANALYSIS

Design

COVID-LIV is a prospective observational cohort study of households in the Liverpool City Region, UK. Cohorts are followed up for an initial period of 12 weeks and then up to three years, observing the incidence of household transmission of SARS-CoV-2 and characterising changes in the immune response over time. For 12 weeks, all household members are requested to perform weekly self-administered throat and nasal swabbing, along with the collection of blood and other clinical samples at different time points of the study. The study started in July 2020 and is expected to continue until September 2023.

There are social science studies linked to this household study, including longitudinal surveys of all these households focusing on the impacts of the pandemic on the residents included in this research. In addition, there will be in-depth qualitative interviews at baseline and three months with a purposive sample of these households, focusing on risk perception beliefs and actions.

Study population

Households are recruited from the large metropolitan Liverpool City Region in North West England, UK. The Liverpool City Region comprises six local authorities and has a population of over 1.5 million people. Almost 50% of its population are categorised as living in the 20% most deprived areas of England.[15]

Recruitment procedure

Households were recruited from the established Liverpool household survey undertaken by the NIHR Collaboration for Leadership in Applied Health Research and Care (CLAHRC, now ARC).[16] The established study framework contains over 7000 households, representing a spectrum of demographic and socioeconomic characteristics. The initial selection process was undertaken by ARC team members with appropriate permission to access the original survey data. Individuals who indicated a willingness to be re-contacted for further research participation were identified and contacted by the COVID-LIV study team about their potential participation in this study.

To supplement the number of recruits, other methods to reach out to potential participants were utilised, which include text messages sent from local general practitioner (GP) surgeries to their patients containing information about the study; and sharing study information through the University of Liverpool media, local media outlets, and the social media of the researchers and stakeholder organisations. Interested households that contacted the study team are recruited if they fulfil the study criteria as follows:

For peer review only

Inclusion criteria

1. All people in the household willing to take part
2. At least one adult within the household must speak English and willing to translate
3. Have provided informed verbal and written consent, or personal legal consent for those lacking capacity
4. Ability and willingness to undertake self-swabbing
5. Intention to be resident for at least six months within their current household – except for students, military personnel, and other professions who may have to move away from home for purposes of study or employment

Exclusion criteria

1. Contraindication to throat and nasal swab or blood sampling
2. One or more members of the household have had a proven COVID-19 test (positive PCR test for SARS CoV-2)
3. No members of the household can speak English

A household comprises those individuals who reside at the household at the date of contact, even if they do not believe this is their primary residence and are intending to stay for at least six months beyond the date of enrolment and first sampling. Regularly attending persons such as carer and cleaner are classified as attendees and are asked to participate in the study, although their participation status does not affect the eligibility of the household. A participant aged 16 years or above is considered an adult.

Participant pathway

This section provides the pathway details of study participant from enrolment up to the end of the study (Figure 1).

Consent procedure

The consent process consists of two phases; an introductory communication, followed by a visit to establish consent and sampling. At the first phase, potential households from the list of contacts from ARC will be contacted by phone or email wherever possible for ease and speed of communication and to minimise transmission risk. The same method of communication applies to participants that directly contacted the study team in response to advertisement through GP surgeries and other forms of media communication. During this phase, potential participants are given a brief explanation about the study, access to information on the study website is confirmed, and any queries are answered.

Following initial contact and expression of interest by the household, a visit arrangement by research nurses is made. During the visit, where every household member is expected to be present, printed information sheets are provided along with further discussions on the purpose of the study and procedures required (see online supplementary appendix file 1). Written informed consent is expected to be provided for each member of the household. In addition to the parent or guardian consent form, children are provided with age-relevant information sheets; assent is obtained if the child is aged 8 - 15 years old and deemed capable of assessing the study documentation provided.

Baseline visit

After written consent forms have been acquired from all household members, a baseline visit date is arranged. The visit is done on the day the consent forms are signed, or another date is arranged if necessary. During the baseline visit, the research nurses collect throat and nasal swab, blood samples (or finger prick sample if not suitable for venepuncture), nasal mucosal sample, and saliva sample from

For peer review only

all adult participants. Only a finger prick sample and saliva sample are collected from children. The research nurses also train the participants on how to perform throat and nasal swabbing themselves. Instruction on the procedure of self-swabbing is given to each household, along with the swab kits for the following weeks.

The first 12 weeks of participation

Participants are instructed to perform self-throat and nasal swab every week for a total of 12 weeks after enrolment; samples are collected by a courier. A questionnaire is sent each week through email or phone call if no email address is provided, requesting information about the participant's health condition and activities from the past seven days. Optional stool samples are collected from consenting participants at weeks 6-8 and 12-14 from enrolment.

Positive swab and result notification

If a positive SARS-CoV-2 swab result occurs during the first 12 weeks of self- throat and nasal swabbing, participants are informed of the result within 72 hours of sample receipt at the laboratory. Positive case details are passed to the National Health Service (NHS) test and trace according to Public Health England statutory requirement. Upon notification, participants are given information on self-isolation and are provided with other relevant guidelines from the UK government and the NHS. The participant's GP is also informed, and additional clinical advice is available from the infectious diseases team at Liverpool University Hospitals NHS Foundation Trust or Alder Hey Children's Hospital NHS Foundation Trust if deemed necessary by the study clinical team.

Following notification, a household visit is arranged to obtain additional samples of blood (or finger prick), throat and nasal swab, nasal mucosal swab, and saliva from the positive case and other household members. The visit is expected to be done within three days following result notification; repeat visits are arranged at 14 days and 12 weeks after result notification. If a participant has more than one positive PCR swab result during the course of the study, this will trigger re-start of the additional sampling schedule if more than six weeks have elapsed from the first positive test in order to identify re-infection.

Follow-up visits

The first follow-up visit is performed at 12 weeks after enrolment for households with and without COVID-19 cases. Samples of blood (or finger prick) are collected from households with no positive case, whilst samples of blood (or finger prick), throat and nasal swab, nasal mucosal swab, and saliva are collected from households with at least one positive case. Repeat visits to households with a history of a positive throat and nasal swab, or seropositivity at baseline or at 12-week follow-up, will be conducted at six months, one year, two years, and three years post enrolment.

For peer review only

Clinical sample and laboratory investigation

The following section provides more detail on the clinical samples that are obtained at different time points during the study (Table 1).

Table 1. Collection of clinical samples for the COVID-LIV study

**Timepoint**	**Baseline / enrolment**	**Week 1 - 12 (Day 8, Day 15 etc.)**	**Weeks 6-8 and 12-14**	**Any time in weeks 0-12**	**Week 12 follow-up**	**Week 12, Month 6, Year 1,2, 3**
Participant group	All participants	All participants	All participants[2]	PCR swab positive participants/household members of swab positive participants[3]	PCR swab negative participants	Seropositive / PCR swab positive participants and their household members
Throat & Nasal Swab	X	X				
Blood samples	X			X	X	X
Stool Sample			X[2]	X		
Nasal mucosal sample	X[1]			X[1]		X[1]
Saliva sample	X			X		X

[1] Adult participant only

[2] Optional - additional consent required

[3] Samples taken at day three and day 14 after PCR swab positive test

Throat and nasal swab

A combined throat and nasal swab are taken for detection of SARS-CoV-2 at baseline by the participant under the guidance from the research nurses. Swabs are then taken by the participants at home and collected on a weekly basis for 12 weeks. This sample is collected using nylon flocked dry swabs placed into plastic tubes and transported to the laboratory to be tested within 72 hours. Swabs are processed for RNA extraction (Zymo) and qPCR (Primer Design Novacyt). Remaining amies medium and extracted nucleic acid will be stored for future virology research.

Virus sequencing

Nucleic acid from positive throat and nasal swabs (and a small number of negative swabs as controls) will be transferred to the Centre for Genomic Research, University of Liverpool for SARS-CoV-2 whole-genome sequencing using the nanopore technology and the ARTIC network protocol (https://www.protocols.io/view/ncov-2019-sequencing-protocol-bbmuik6w).

Blood sample

Up to 60 ml of whole blood are collected from each adult participants (or finger prick sample, if unsuitable for venepuncture). Children under the age of 16 years will have finger prick and blood spot collection rather than venepuncture, and a smaller amount of blood collected.

For peer review only

The baseline and 12-week follow-up blood samples will be used to determine the prevalence of exposure to SARS-CoV-2 at a certain point of the epidemic. These data will be used to supplement virology data to maximise the identification of SARS-CoV-2 exposure.

Peripheral blood mononuclear cells (PBMCs) will be isolated from different time points of infection using Ficoll density centrifugation. Briefly, blood collected from sodium heparin tubes will be placed on a Ficoll cushion and centrifuged to retrieve PBMCs. Cells will then be washed and frozen down in 90% fetal bovine serum (FBS) and 10% DMSO for downstream assays.

Antibody responses

The antibody response will be measured over time at baseline, 12 weeks, 6 months, and 1-, 2- and 3-years post-infection by ELISA, pseudo-virus neutralisation and SARS-CoV-2 neutralisation in a subset. The proportion of participants positive at each time point will be determined, and the magnitude of antibody titres measured. If positive cases are detected, neutralising antibody titres will be measured, and virus isolation will be attempted allowing testing of the serum neutralising capacity against the actual virus infecting the participant. Mucosal antibody and cytokine responses will be tested. These experiments will determine whether serum antibody measurements correlate with mucosal antibody and whether either is an adequate correlate of immunity. Parallel samples from household contacts (who are highly likely to be exposed) will also be collected and studied in order to determine what, if any, factors protect against the acquisition of infection, or correlate with sterilising immunity.

T cell responses

Antigen-specific responses will be measured following *ex-vivo* stimulation with SARS-CoV-2 peptide pools. PBMCs isolated at the various time points will be stimulated with various peptide pools and ICS (intracellular cytokine stain) and activation marker assays will be performed to characterise the SARS-CoV-2 T cell responses. Single-cell RNA-seq assays will also be done to explore the breadth of the T cell response to determine qualitative differences in the T cell repertoire. Where sample allows, T cell epitopes will be mapped using a synthetic peptide library and tested for cross-reactivity against common cold coronaviruses.

Innate response

Whole blood stored in RNA stabilisation solution (tempus tubes) will be subjected to RNA isolation and sequencing to characterise the innate immune response. These data will inform and refine the above experiments and have the potential to be related to the ISARIC 4C dataset (hospitalised severe cases) as a mild disease group.

Genomic testing

HLA (human leukocyte antigen) typing will be undertaken along with characterisation of other important immune mediating characteristics, e.g. Angiotensin reception 2 (ACR2).

Stool sample

Stool samples are collected from adult participants who test positive from a PCR swab and from their consenting household contacts at approximately 3 and 14 days after confirmation of a positive PCR. Samples are transported to the University of Liverpool where they are frozen down for downstream assays, including for SARS-CoV-2 sequencing. Additionally, optional stool samples are requested from all participants at two time points from their enrolment; at approximately week 6 – 8 and week 12 – 14.

For peer review only

Nasal mucosal and saliva sample

At the baseline visit, nasal mucosal and saliva samples are collected from adult participants and all participants, including children, respectively. The nasal mucosal sample is collected using synthetic absorptive matrix (SAM) strips, and saliva sample is collected using an oral fluid collection device, both are collected for antibody analyses. Additional samples are also collected from adult participants who subsequently tested positive from PCR swab and their household contacts.

Outcome measures

Primary endpoints

1. Incidence of paucisymptomatic and asymptomatic SARS-CoV-2 infections index cases, including the prevalence of infection or past infection at baseline serology status
2. Incidence of secondary household cases
3. Risk factors for household transmission

Secondary endpoints

1. Analysis of household contact patterns
2. Description of clinical phenotypes of the index cases
3. Genomic characterisation of SARS-CoV-2
4. Characterisation of the immune response in index cases and exposed household contacts
5. Prevalence of SARS-CoV-2 household faecal shedding

Data analysis

The results of the analyses will be reported according to the STROBE guidelines.[17] This will include a descriptive analysis of households; paucisymptomatic and asymptomatic primary household index cases; and secondary household cases.

Environmental, demographic, and behavioural risk factors for secondary transmission among household contacts of symptomatic and laboratory-confirmed cases of COVID-19 will be investigated. Cases in households will be ordered by date of symptom onset. The first symptomatic COVID-19 case in the household will be classified as the probable household index case. Secondary COVID-19 cases will be defined as any COVID-19 case with an onset of illness within seven days following the onset of the index case.

The primary attack rate will be calculated as the number of households with a primary case divided by the total number of households in the study. The household secondary attack rate will be calculated from the number of households with at least one secondary COVID-19 case divided by the total number of households at risk. The individual household members attack rate will be calculated from the number of household members with secondary COVID-19 illness divided by the total number of household members at risk.

Index cases will be described in relation to demographics, employment, and contact history. A risk factor analysis will be undertaken to investigate variables associated with secondary attack cases within households. Risk factors will include data on contacts, viral load measurements, household characteristics, and other variables that emerge in external reports or literature that may be linked with transmission.

Data and statistical analysis of serology and other immunological parameters will be done using GraphPad Prism, FlowJo v10, R and other bioinformatics software.

For peer review only

Sample size

The study should be regarded as exploratory. The initial constraints on sample size are primarily access to testing on a weekly basis. Referring to the current data on the secondary attack rate (SAR) of 10.5 – 45% of contacts with a hazard ratio of 1.5 or 2.0, using a single sample Cox proportional hazard model with 80% power and 10% study withdrawal, we propose an initial sample size of 300 households, which will contain approximately 1000 individuals.[18–20]

ETHICS AND DISSEMINATION

The study has received approval from the NHS Research Ethics Committee; REC Reference: 20/HRA/2297, IRAS Number: 283464. Protocol amendments have been and will be reported to the Research Ethics Committee, as will any serious adverse events. The study participants are informed that all data collected are for research purposes only and that they have the right to withdraw from the study at any time.

PATIENT AND PUBLIC INVOLVEMENT

The protocol has been reviewed by the patient and public involvement (PPI) committee of the Institute of Infection & Global Health. The study design, participant acceptability, and perceptions have been reviewed and discussed. The necessary speed to get this work up and underway has prevented a more standard input from the PPI group. We will report test results to participants in plain language.

PROJECT GOVERNANCE

The study is coordinated by the Liverpool Clinical Trials Centre. A study steering group has been established to enable effective achievement of the project objectives. The steering group includes representatives from academia, public health, and lay membership.

DISSEMINATION OF RESEARCH FINDINGS

The findings will be presented at professional and scientific conferences. The results will also be published in peer-review publications and if appropriate, published first as pre-prints to enable a timely public health response to COVID-19. Interim and final reports will be submitted to the funders and the steering group. We also work with our institute PPI panel to identify and produce materials to disseminate to the general public, including study participants.

DISCUSSION

COVID-LIV will demonstrate the role of household transmission of SARS-CoV-2 in a cohort of households in the Liverpool City Region, UK. By observing households with no apparent previous infection of COVID-19, it is hoped that the incidence, determinants of transmission, and contribution

For peer review only

of paucisymptomatic and asymptomatic cases can be described, filling a knowledge gap in how the disease transmits within the population in the Liverpool City Region. Characterising immune responses in a cohort of mild infections will provide a better understanding of how natural infection alters immune parameters over time, allowing a better understanding of immunity against COVID-19 infection that may help inform vaccine development and delivery.

Strengths

COVID-LIV aimed to recruit a cohort of households across a representative range of socioeconomic status in the Liverpool City Region. The household cohort allows for identification of paucisymptomatic and asymptomatic COVID-19 cases, which will provide a better representation of the impact of COVID-19 in the community and extent of transmission through the sampling of high probability exposed household members. The prospective nature of the study allows the determination of a true incidence rate and risk factors for SARS-CoV-2 transmission with less recall bias. The longitudinal study design enables the analysis of the immunological response and faecal shedding of SARS-CoV-2 during different stages of the disease. It also allows observation of the natural progression of mild cases from a pre-infection stage sample collection to allow the interrogation of T cell repertoires and their association with acute infection.

Limitations

The cohort households may be biased by those that are most engaged with COVID-19 and disease control, leading to a low level of secondary infections as participating individuals are more likely to take precautions against transmission. Low level of detectable infections may also be observed due to the study observation across different seasonality time points. Reliance on repeated self-sampling may lead to diagnostic inconsistencies, although instructions were given, and techniques were carefully assessed by the research nurses during the initial baseline visit. Exclusion of non-English speaking families may exclude potential high-risk households resulting in under detection of incidence rate and more severe cases.

ACKNOWLEDGEMENT

The authors would like to thank and appreciate all the participants in the study for their invaluable contribution and the external advisory panel members: Jonathan M. Read, Antonia Ho, and Cliona McDowell.

The following are members of the COVID-LIV Study Group:

Principal investigator: Neil French

Study Investigators: Lance Turtle, Daniel Hungerford, Krishanthi Subramaniam, Roberto Vivancos, Mark Gabbay, Iain Buchan, Enitan D. Carrol, Miren Iturriza-Gomara, Tom Solomon, Nigel A. Cunliffe, Emily Adams, Carrol Gamble

Lay members: Lynnette Crossley, Neil Joseph

Fieldwork team: Wega Setiabudi, Marcella Vaselli, Moon Wilton, Lee D. Troughton, Samantha Kilada, Katharine Abba, Victoria Simpson, John S.P. Tulloch, Lynsey Goodwin, Rachael Daws, Shiva Seyed Forootan, Susan Dobson, Rachel Press, Vida Spaine, Lesley Hands, Kate Bradfield, Carol McNally

Project management: Tracy Moitt, Silviya Balabanova, Chloe Donohue, Lynsey Finnetty, Laura Marsh

For peer review only

Clinical and laboratory team: William Greenhalf, Dean J. Naisbitt, Victoria E. Shaw, Stephen Aston, Gareth Platt, Paul J. Thomson, Monday Ogese, Sean Hammond, Kareena Adair, Liam Farrell, Joshua Gardner, Kanoot Jaruthamsophon, Serat-E Ali, Adam Lister, Laura Booth, Milton Ashworth, Katie Bullock, Benjamin W.A. Catterall, Terry Foster, Lara Lavelle-Langham, Joanna Middleton, William Reynolds, Emily Cass , Alejandra Doce Carracedo, Lianne Davies , Lisa Flaherty, Melanie Oates , Nicole Maziere, Jennifer Lloyd, Christopher Jones, Hannah Massey, Anthony Holmes, Nicola Carlucci, Vanessa Brammah, Yasmyn Ramos, Daniel Allen, Jane Armstrong, Debbie Howarth, Eve Wilcock, Jena Lowe, Jayne Jones, Paula Wright, Iain Slack, Simone McLaughlin, Jessica Mason, Thomas Edwards, Claudia McKeown, Elysse Hendrick, Chris Williams , Rachel Byrne , Kate Buist, Gala Garrod, Sophie Owen

Statisticians: Ashley Jones, Efstathia Gkioni

For peer review only

AUTHOR'S CONTRIBUTION

Contributors: NF, NAC, DH, LT, MIG conceived of the study. DH, KS, NF, NAC, LT, MIG, TS, SA, IB, MG, RV, MW, MV, WS, EDC, ERA initiated study design and protocol development. LC and NAC contributed to protocol development. GP, VES, WG, DJN, TM, LDT, SK, MW, KA, VS, WG, JSPT, LG, RD, SSF, SD, RP, VS, LH, KB, CM, PJT, MO, SH, KA, LF, JG, KJ, S-EA, AL, LB, MA, KB, BWAC, TF, LL-L, JM, WR, EC, ADC, LD, LF, MO, NM, JL, CJ, HM, AH, NC, VB, YR, DA, JA, DH, EW, JL, JJ, PW, IS, SMcL, JM, TE, CMcK, EH, CW, RB, KB, GG, SO, LF, LM helped with implementation. DH, NF, AJ, EG, CG, EM provide statistical expertise in statistical design and have produced the analysis plan. TM, SB, CD provided study management and research support. WS and DH drafted the manuscript. All authors contributed to refinement of the study protocol.

FUNDING STATEMENT

This study is co-funded by the National Institute for Health Research Health Protection Research Unit (NIHR HPRU) in Gastrointestinal Infections, a partnership between Public Health England, the University of Liverpool and the University of Warwick; the NIHR HPRU in Emerging and Zoonotic Infections, a partnership between Public Health England, the University of Liverpool in collaboration with the Liverpool School of Tropical Medicine and the University of Oxford; the Centre of Excellence in Infectious Disease Research (CEIDR); and the Alder Hey Charity. Grant number for the fundings is not applicable.

NF is funded by the National Institute for Health Research Health Protection Research Unit (NIHR HPRU) in Emerging and Zoonotic Infections, the Centre of Excellence in Infectious Diseases Research (CEIDR), and the Alder Hey Charity. We also acknowledge the support of Liverpool Health Partners and the Liverpool-Malawi-Covid-19 Consortium.

This research was funded in whole, or in part, by a Wellcome Trust fellowship awarded to LT [205228/Z/16/Z]. For the purpose of Open Access, the author has applied a CC BY public copyright licence to any Author Accepted Manuscript version arising from this submission. LT is also supported by the National Institute for Health Research Health Protection Research Unit (NIHR HPRU) in Emerging and Zoonotic Infections (NIHR200907) at University of Liverpool in partnership with Public Health England (PHE), in collaboration with Liverpool School of Tropical Medicine and the University of Oxford. LT is based at University of Liverpool.

WS is funded by the Ministry of Finance, the Republic of Indonesia through the Indonesia Endowment Fund for Education (Lembaga Pengelola Dana Pendidikan or LPDP) scholarship for doctoral study (201807220413052).

DH is funded by a National Institute for Health Research (NIHR) Post-doctoral Fellowship (PDF-2018-11-ST2-006).

KS is funded by a HEFCE-funded University of Liverpool Tenure Track Fellowship.

EDC acknowledges funding from the NIHR i4i Programme (II-LA-0216-20002), HTA Programme (15/188/42, 17/136/13), EME Programme (NIHR129960) and H2020 (Project No. 848196).

MG is part-funded by the NIHR Applied Research Collaboration North West Coast.

The views expressed are those of the author(s) and not necessarily those of the NHS, the NIHR, the Department of Health and Social Care, Public Health England, or other funding bodies.

COMPETING INTERESTS STATEMENTS

MW is funded under a grant from Astra Zeneca and University of Liverpool for an unrelated project.

REFERENCES

1. Walker PG, Whittaker C, Watson O, et al. The Global Impact of COVID-19 and Strategies for Mitigation and Suppression. *Imperial College COVID-19 Response Team*. 2020;March(June):19. doi.org/10.25561/77735.

2. Coronavirus disease (COVID-19). https://www.who.int/emergencies/diseases/novel-coronavirus-2019. Accessed November 3, 2020.

3. Boulware DR, Pullen MF, Bangdiwala AS, et al. A Randomized Trial of Hydroxychloroquine as Postexposure Prophylaxis for Covid-19. *New England Journal of Medicine*. 2020;383(6):517-525. doi:10.1056/nejmoa2016638

4. UK medicines regulator gives approval for first UK COVID-19 vaccine - GOV.UK. https://www.gov.uk/government/news/uk-medicines-regulator-gives-approval-for-first-uk-covid-19-vaccine. Accessed December 16, 2020.

5. Coronavirus: The world in lockdown in maps and charts - BBC News. https://www.bbc.co.uk/news/world-52103747. Accessed November 3, 2020.

6. Flaxman S, Mishra S, Gandy A, et al. Estimating the effects of non-pharmaceutical interventions on COVID-19 in Europe. *Nature*. 2020;584(7820):257-261. doi:10.1038/s41586-020-2405-7

7. The Global Economic Outlook During the COVID-19 Pandemic: A Changed World. https://www.worldbank.org/en/news/feature/2020/06/08/the-global-economic-outlook-during-the-covid-19-pandemic-a-changed-world. Accessed November 3, 2020.

For peer review only

8. McGinty EE, Presskreischer R, Han H, Barry CL. Psychological Distress and Loneliness Reported by US Adults in 2018 and April 2020. *JAMA - Journal of the American Medical Association*. 2020;324(1):93-94. doi:10.1001/jama.2020.9740

9. Sachs JD, Abdool Karim S, Aknin L, et al. Lancet COVID-19 Commission Statement on the occasion of the 75th session of the UN General Assembly. *The Lancet*. 2020;396(10257):1102-1124. doi:10.1016/S0140-6736(20)31927-9

10. Madewell ZJ, Yang Y, Longini IM, Halloran ME, Dean NE. Household Transmission of SARS-CoV-2: A Systematic Review and Meta-analysis. *JAMA Network Open*. 2020;3(12):e2031756. doi:10.1001/jamanetworkopen.2020.31756

11. Stay at home: guidance for households with possible or confirmed coronavirus (COVID-19) infection - GOV.UK. https://www.gov.uk/government/publications/covid-19-stay-at-home-guidance/stay-at-home-guidance-for-households-with-possible-coronavirus-covid-19-infection. Accessed December 15, 2020.

12. Koh WC, Naing L, Chaw L, et al. What do we know about SARS-CoV-2 transmission? A systematic review and meta-analysis of the secondary attack rate and associated risk factors. Leekha S, ed. *PLOS ONE*. 2020;15(10):e0240205. doi:10.1371/journal.pone.0240205

13. Liverpool City Region to move into “very high” local COVID Alert Level following rise in coronavirus infections - GOV.UK. https://www.gov.uk/government/news/liverpool-city-region-to-move-into-very-high-local-covid-alert-level-following-rise-in-coronavirus-infections. Accessed November 5, 2020.

14. Liverpool to be regularly tested for coronavirus in first whole city testing pilot - GOV.UK. https://www.gov.uk/government/news/liverpool-to-be-regularly-tested-for-coronavirus-in-first-whole-city-testing-pilot. Accessed December 16, 2020.

15. Liverpool City Council. The Index of Multiple Deprivation 2019 A Liverpool analysis Executive Summary. 2019. https://liverpool.gov.uk/media/1359213/imd-2019-liverpool-analysis-main-report.pdf.

16. Giebel C, McIntyre JC, Daras K, et al. What are the social predictors of accident and emergency attendance in disadvantaged neighbourhoods? Results from a cross-sectional household health survey in the north west of England. *BMJ Open*. 2019;9(1):e022820. doi:10.1136/bmjopen-2018-022820

17. von Elm E, Altman DG, Egger M, Pocock SJ, Gøtzsche PC, Vandenbroucke JP. The Strengthening the Reporting of Observational Studies in Epidemiology (STROBE) Statement: Guidelines for Reporting Observational Studies. *PLoS Medicine*. 2007;4(10):e296. doi:10.1371/journal.pmed.0040296

18. Liu Y, Eggo RM, Kucharski AJ. Secondary attack rate and superspreading events for SARS-CoV-2. *The Lancet*. 2020;395(10227):e47. doi:10.1016/S0140-6736(20)30462-1

19. Burke RM, Midgley CM, Dratch A, et al. Active Monitoring of Persons Exposed to Patients with Confirmed COVID-19 — United States, January–February 2020. *MMWR Morbidity and Mortality Weekly Report*. 2020;69(9):245-246. doi:10.15585/mmwr.mm6909e1

20. Bi Q, Wu Y, Mei S, et al. Epidemiology and transmission of COVID-19 in 391 cases and 1286 of their close contacts in Shenzhen, China: a retrospective cohort study. *The Lancet Infectious*

For peer review only

Diseases. 2020;20(8):911-919. doi:10.1016/S1473-3099(20)30287-5

FIGURE LEGENDS

Figure 1: Participant pathway

For peer review only

Figure 1. Participant pathway

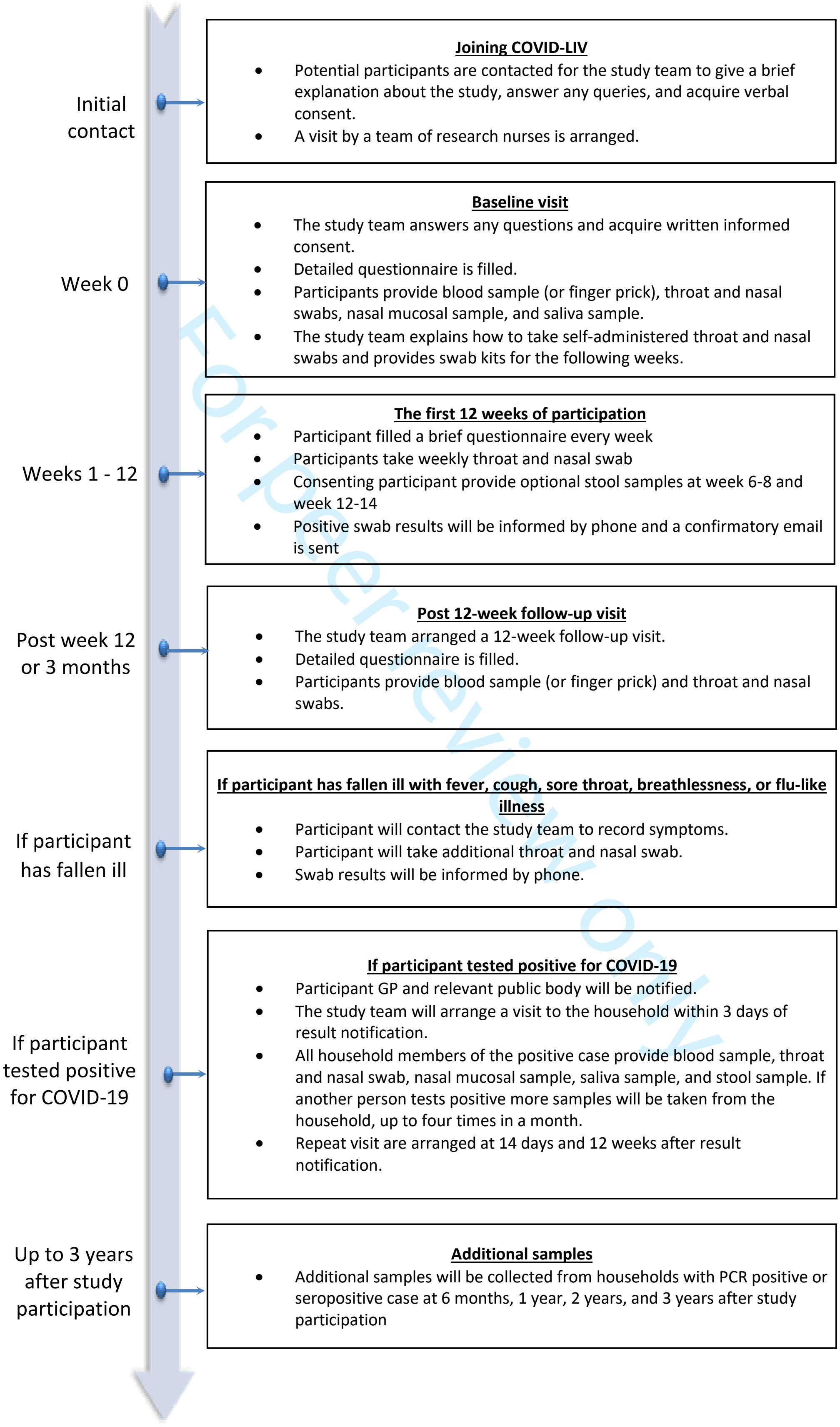

For peer review only - http://bmjopen.bmj.com/site/about/guidelines.xhtml

COVID-LIV Study

UNIVERSITY OF LIVERPOOL

Liverpool Household COVID-19 Cohort Study (COVID-LIV)

Adult Information Sheet for COVID-LIV

- You were contacted by telephone or you have contacted us and verbally agreed to take part in a research study called COVID-LIV. You also provided information about yourself.
- Please now take time to read the following information carefully (note, you may have received this information via email following the telephone call). **Part 1** tells you the purpose of the study and what will happen to you if you take part. **Part 2** gives you more detailed information about the conduct of the study.
- You can ask a member of the research team if there is anything that is not clear, or if you would like more information.
- Taking part is voluntary. If you don't want to take part then you don't need to give a reason.
- If you want to take part but other members of the household do not want to, we may not be able to include you in the study.
- The COVID-LIV study team are studying how COVID-19 spreads in the community, inside households.
- This is being done using swab tests – the same tests that you would have if you came into hospital with COVID-19. If you choose to, you can also provide a stool sample at 6-8 and 12-14 weeks.
- We need all kinds of different households across Liverpool city region to take part.
- The study is currently funded for 12 months whilst additional funding is obtained to continue for a further three years.

How to contact the study team:

If you have any questions about this study, please talk to a member of the study team who visits you, or call: ###

Professor Neil French is the lead Investigator.

Contents:

Part 1 – Purpose of the study and what will happen if you take part

- Why are we doing the COVID-LIV study?
- Why have I been chosen?
- Do I have to take part?
- What will happen to me if I take part?
- What will I have to do if I take part?
- What are the benefits and risks of taking part?
- What happens if I change my mind?
- What happens when the study stops?
- What if there is a problem?
- Will my taking part in the study be kept confidential?

Part 2 – Detailed information about the conduct of the study

- Who is running the study?
- How will my information be collected and handled?
- What are my choices about how my information is used?
- Information sharing for other research
- Where can I find out more about how my information is used?
- What will happen to the nose and throat swabs, and the blood, saliva and stool samples I give?
- What if there is a problem?

If you become ill or suspect you have COVID-19 please follow government guidelines

LIVERPOOL CLINICAL TRIALS CENTRE LCTC

For peer review only - http://bmjopen.bmj.com/site/about/guidelines.xhtml

PART 1: Purpose of the study and what will happen if you take part

Why are we doing the COVID-LIV study?

COVID-19 (or COVID for short) is the name of the disease that is caused by the new coronavirus. This coronavirus was first found in China in January 2020. The new coronavirus has since spread all round the world. COVID-19 is caused by a virus strain called SARS-CoV-2. We want to understand what factors determine transmission of the virus and how our bodies respond and become resistant to it.

Most people who get COVID-19 will be fine as it is not a serious disease in most people. Some people might not even know that they had it. But in a few people, COVID-19 makes them very sick. They may need a ventilator to help them breathe, or may even die. In order to prevent this, the government has asked everyone except essential workers to stay at home whenever possible. Whilst this will work, it has other effects, for example preventing people from working, so there is a great cost to the country, and to our personal freedoms. We need to understand how COVID-19 spreads, so we can help tell the government when to stop advising people to stay home, and what might happen as they do tell us we can go out again.

The results from this study will be used to provide valuable information for the government and local public health to plan the next stage of the COVID-19 response – that is how we step down from the lock down and back to more contacts and interactions.

Why have I been chosen?

You, and your household, have either been selected because you were part of another study before, called CLAHRC NWC Household Health Survey or you have responded to one of our communications seeking volunteers. Those in the household study gave permission to be re-contacted again about other studies. Therefore, we are contacting you to ask if you would like to take part in this study.

We are selecting different types of households (for example with different numbers of people, or those with and without children) to take part in COVID-LIV. We need all different kinds to take part so we have not approached you based on anything particular about you or your household, or family. We are looking for around 1000 people from 300 different households to take part, so that we can be sure the people in the study are just like those in the whole community.

Do I have to take part?

No, taking part is voluntary. It is up to you to decide whether or not you want to take part.

We also want all the members of the household to take part. If you want to but other members of the household do not want to participate we may not be able to include you.

If you do decide to take part, we will ask you to sign a consent form.

If you decide to take part, you can also choose to stop at any time without giving a reason.

The decision you make on whether to take part or not will not affect the care you receive for COVID-19 now, or in the future.

What will happen to me if I take part?

After you have signed the consent form, we will ask you to complete a questionnaire (we will ask you about members of your household, diet, employment, and some other things). The researcher will take up to 60ml blood sample from you. This sounds like a lot, but it is in fact only about 1½ egg cups in size. For the participants unable to donate blood, the researcher will take a finger prick test and blood spot collection. We will also collect a sample of saliva with a mini sponge and a sample of nasal fluid with a small piece of special filter paper. We will then explain how you can take nose swabs from yourself or other members of the household and ask you to do the first swab in the presence of a researcher to check you are able to do it correctly. These swabs are for COVID-19 testing, and are easy to use. If you feel ill at the time of the first visit we will only take the nose swab to see if you have COVID19 and arrange a further visit to collect blood and other samples depending on the swab result. You will swab your nose once a week, for 12 weeks (or at any other time if you think you have fallen

For peer review only

For peer review only - http://bmjopen.bmj.com/site/about/guidelines.xhtml

ill with fever, cough, sore throat, breathlessness or flu-like symptoms). We will phone you, or send a text message, to remind you to take the nose swabs once a week. If you struggle to take the nose swabs yourself, simply let us know and we can arrange for the researcher to visit you and help take the swabs.

We will let you know when a courier will collect the swabs from you – this will happen on a weekly basis. The swabs will be taken to a laboratory team who will do COVID-19 tests on them. This takes up to three days, and we will phone you, or send a text message, with the results.

After the first 12 weeks, we do not need swabs every week. However, we would still like you to collect nose swabs at any point in the study if you think you have fallen ill with fever, cough, sore throat, or breathlessness. At this point, the processing of swabs may change depending on local NHS testing policy

The researcher will visit and collect up to 60ml blood samples after you have been in the study for 12 weeks (3 months), then again at 6 months, 1 year, 2 years and 3 years after you started the study. For the participants unable to donate blood, the researcher will take a finger prick test and blood spot collection. At these visits, you will also complete a questionnaire.

If a nose swab has a positive test result: *We will provide support in terms of your healthcare (either via the phone, or face-to-face), and we will also visit you a few days after your positive result, and then 2 weeks after that. We will inform your GP of your positive result. Public Health England maybe informed in line with their current guidelines. When we visit, we will ask you more questions, and take some more samples, both from the person in the household who has tested positive, and from the rest of the household as well. The samples we will take are: another nose swab, a swab for saliva, a stool sample, and further blood samples (up to 60ml). We may also provide you with a special device for rapid diagnosis to take a nose/throat swab, but this is only if it is approved for use and part of NHS guidelines. If another member of the household tests positive, then we will take additional blood, saliva, nose and throat samples off them as well. The maximum number of times we take samples would be about 4 in a month.*

If, at any point, you become ill or suspect you have COVID-19, you can call the telephone number given on the first page of this information sheet. If you suspect you have COVID-19 and need to talk to someone outside of usual office hours, you should call **NHS ###.**

The study is planned to last for three years. At the moment the study is going to be started for the first 12 weeks (3 months); continuing the study after that will depend on the study team obtaining more funding. However, our intention is to run the study for three years, so we are asking for permission for this up front from you, so that we do not need to keep coming back to you.

We will let you know when the study ends.

What will I have to do if I take part?

If you agree to take part, you will be asked to sign a consent form. You will be provided with a copy of the consent form and the information sheet to keep.

Once you have signed the consent form, you will be asked to follow the study plan (see study timeline diagram below).

You will have to:

- Provide swab of the throat and nose at the beginning, repeated weekly for the first 12 weeks and at any time point you have fallen ill with fever, cough, sore throat, or breathlessness taken by yourself
- Be ready at pre-set timepoints for the courier to collect the swabs (once a week)
- Provide blood samples (or finger prick test, if unable to donate blood)
- Provide saliva swabs (only if you test positive for COVID-19)
- Provide stool sample at week 6-8 and 12-14 (optional), and if you test positive for COVID-19)
- Complete questionnaires (either by yourself or over the telephone)
- Provide information on your health and wellbeing

For peer review only

For peer review only - http://bmjopen.bmj.com/site/about/guidelines.xhtml

Initial contact

Joining COVID-LIV

- Potential participants are contacted for the study team to give a brief explanation about the study, answer any queries and acquire verbal consent.
- A visit by a team of research nurse or doctor is arranged.

Week 0

Initial visit

- The study team will answer any questions and acquire written informed consent.
- Detailed questionnaire will be filled
- Participants will provide blood sample (or fingerprick), nose and throat swabs, nasal strip and saliva.
- The study team will explain how to take self-administered nose swabs and provide with swab kits for the next weeks

Weeks 1 - 11

Weekly questionnaires and swabs

- Participant will fill a brief questionnaire
- Participants will take weekly nose swab
- Participant will provide an optional stool samples at week 6-8.
- Courier will come and collect the samples every week.

Week 12

Questionnaire, blood sample and swabs

- You will complete a detailed questionnaire.
- If you choose to, you can also provide a stool sample at week 12-14.
- We will take a blood sample (or fingerprick), and a nose swab.
- We will phone/text you with the results in 2/3 days.

If you test positive (study nurse visits you)

If you test positive for COVID-19 (this visit will occur a few days later, and again about 2 weeks after that)

We will inform your GP of the positive result.
We will ask some questions about your health.
We will take a blood sample, nose swab, throat swab, nasal strip, a swab for saliva and a stool sample from all members of the household where someone has tested positive. If another person tests positive more samples will be taken off the household, up to 4 in a month.
Also at 3, 6, 12, 24 and 36 months.
We may also take a nose/throat swab using a special device, but this is only if it is approved for us to use.

If additional funding is received, and the study continues

If the study continues past 3 months, this visit will occur 6 months, 1 year, 2 years and 3 years after you consented to join the study

You will complete a detailed questionnaire.
We will take a blood sample.

12 weeks, or up to 3 years, after you join the study

End of your participation

No further visits/samples/data collection will occur once you have reached this point, unless additional funding is received.

For peer review only

For peer review only - http://bmjopen.bmj.com/site/about/guidelines.xhtml

What are the benefits and risks of taking part?

The main benefit is that you will know if you have had COVID-19 or not. If you have symptoms, and the test is negative, we will tell you this. This means that, once you have **self-isolated for 7 days** (as per government advice at the beginning of June, if this changes we will let you know), you could still go out to work or to the shops because we will know for sure that you have not got COVID-19. We will also be doing antibody tests on your blood, so you will be tested for immunity as well. However, at the moment we are not sure if these tests means that you will actually be protected from repeat infections in the future. Studying that is one of the aims of the study.

The risks are minor bruising from the blood samples taken, and researchers entering your house. However, the researchers will be wearing Personal Protective Equipment (PPE) at all times, to prevent transfer of COVID-19.

What happens if I change my mind?

If at any point you decide to stop taking part in the study, you can let a member of the research team know. In order for us to understand why participants withdraw from the study, we may ask you why you have decided to withdraw. However, you do not have to give a reason, if do not want to.

If you do decide to stop taking part, this will not affect your current and future medical care, and your legal rights will not be affected in any way.

Information on how we will handle your information in the event of you withdrawing is detailed in Part 2 of this Information Sheet.

What happens when the study stops?

It is intended that the results of the study will be presented at conferences and published in medical journals so that we can explain to the medical community what our research results have shown. Confidentiality will be ensured at all times and you will not be identified in any publication.

Any information derived directly or indirectly from this research, as well as any patents, diagnostic tests, drugs, or biological products developed directly or indirectly as a result of this research may be used for commercial purposes. You have no right to this property or to any share of the profits that may be earned directly or indirectly as a result of this research. However, in signing this form and donating swabs and blood samples for this research, you do not give up any rights that you would otherwise have as a participant in research.

What if there is a problem?

If, at any point, you become ill or suspect you have COVID-19, you can call the telephone number given on the first page of this information sheet. If you suspect you have COVID-19 and need to talk to someone outside of usual office hours, you should call **NHS ###**.

Any complaint about the way you have been dealt with during the study or any possible harm you might suffer will be addressed. Detailed information is given in Part 2 of this information sheet.

Will my taking part in the study be kept confidential?

Yes. All the confidential information about your participation in this study will be kept confidential. Detailed information on this is given in Part 2.

For peer review only

For peer review only - http://bmjopen.bmj.com/site/about/guidelines.xhtml

PART 2: Detailed information about the conduct of the study

Who is running the study?

University of Liverpool is the Sponsor of this study and is responsible for managing it. They are based in United Kingdom. They have asked that the day to day running of the study is carried out by a team based at the Liverpool Clinical Trials Centre (LCTC, part of the University of Liverpool), and the samples you provide will be managed by members of a laboratory team at the University of Liverpool and Liverpool School of Tropical Medicine (LSTM).

The study has been reviewed by the National Research Ethics Service Committee to make sure that the study is scientifically and ethically acceptable.

This study is funded by the National Institute for Health Research (NIHR) Health Protection Research Unit in Emerging and Zoonotic Infections (HPRU-EZI), Centre of Excellence in Infectious Diseases Research (CEIDR) at University of Liverpool in partnership with Public Health England (PHE), Alder Hey Charity and in collaboration with Liverpool School of Tropical Medicine The views expressed are those of the author(s) and not necessarily those of the NIHR or the Department of Health and Social Care.

We will not receive any payment for including you in this study.

How will my information be collected and handled?

University of Liverpool is the Data Controller for this study and will need to use information from this research project.

This information will include your name, initials, date of birth, contact details, postcode and your NHS number (we will request this from your GP). People will use this information to do the research or to check your records to make sure that the research is being done properly.

Individuals from University of Liverpool, the LCTC, LSTM, and relevant regulatory organisations may look at your research records to check the accuracy of the research study.

People who do not need to know who you are will not be able to see your name or contact details. Your data will have a code number instead.

Data will be sent from the University of Liverpool researchers and LSTM to the LCTC. Data may also be sent from your GP to University of Liverpool researcher, who will then send this to LCTC.

We may notify your GP that you are taking part in the study, and if you test positive for COVID-19, for their information.

We will keep all information about you safe and secure.

Once we have finished the study, we will keep the data for a minimum of 10 years, so we can check the results. We will write our reports in a way that no-one can work out that you took part in the study.

What are my choices about how my information is used?

You can stop being part of the study at any time, without giving a reason, but we will keep information about you that we already have.

We need to manage your records in specific ways for the research to be reliable. This means that we won't be able to let you see or change the data we hold about you.

Information sharing for other research

When you agree to take part in a research study, the information about your health and care may be beneficial to researchers running other research studies in this organisation and in other organisations. These organisations may be universities, NHS organisations or companies involved in health and care research in this country or abroad. Your information will only be used by organisations and researchers to conduct research in accordance with the UK Policy Framework for Health and Social Care Research, or equivalent standards.

If you agree to take part in this study, you will have the option to take part in future research using your data saved from this study.

For peer review only

For peer review only - http://bmjopen.bmj.com/site/about/guidelines.xhtml

Where can I find out more about how my information is used?

You can find out more about how we use your information:

- at the LCTC website: www.lctc.org.uk/privacy
- at LSTM website: https://www.lstmed.ac.uk/lstm-privacy-statement
- at www.hra.nhs.uk/information-about-patients
- in the Health Research Authority leaflet available from www.hra.nhs.uk/patientdataandresearch
- by contacting the University of Liverpool Data Protection Officer on LegalServices@liverpool.ac.uk
- by asking one of the research team
- by sending an email to COVID-LIV-FWCom@liverpool.ac.uk, or
- by ringing us on ###

What will happen to the nose and throat swabs, and the blood, saliva and stool samples I give?

Samples collected for use in COVID-LIV

We will use your samples to test for your body's response to the COVID-19 virus. This will be able to tell us whether you have had the infection with no symptoms, in some cases. We will test your blood to see if you have been exposed before to other coronaviruses.

Your samples will be sent to laboratories at the University of Liverpool and LSTM for analysis. These samples will be coded and the researchers carrying out tests on the samples will not be given information they do not need to carry out the tests and analyse the results. Coded is not the same as anonymous. The study ream will need to know who the sample came from to inform you of the results. It will be possible to use the codes to identify that a result is from your sample. However, once we have given you your results, we do not plan to do this unless there is a good reason to do so. We will maintain this information so that we can properly manage the samples. For instance, sometimes we may need to update our record of your clinical details to help us interpret the results of tests.

If you or a member of your household tested positive for COVID-19: We will use your samples for much more detailed and complex tests on the body's response to the COVID-19 virus. We will study the body's response for everyone in the household – this will help understand how people become infected and how some people may resist the virus. We may also test your blood with other viruses as controls, such as the glandular fever virus, flu, and some other common cold viruses. We will look in detail at the responses of specialised white blood cells in your blood called lymphocytes.

We may store some of your blood, or the cells from your blood for up to 25 years after this research has finished. We may also use some of your cells to make what are called "cell lines" – these are cells that can be kept alive in the lab for a long time (maybe forever) and are used to make it easier to detect and study the lymphocyte responses we are interested in.

We will extract DNA to look at your genes by sequencing the whole of your genome. This will help us understand whether certain genes are related to your ability to fight off the COVID-19 coronavirus. This will include some of the genes unique to your individual immune system, called HLA. These are the same tests that are done before organ transplants, and they are used to tailor our research to each person. There will be left over DNA after we have done this, which would be stored, like the cells from your blood, for up to 25 years after the research has finished.

Samples collected for Future Research

We would like your permission to do other research on these stored samples in the future. This would include looking at factors which are involved in fighting off coronaviruses, and other controls for our experiments such as herpes viruses (like glandular fever), flu, and enteroviruses (e.g. common cold viruses), and other common human viruses. We cannot say now all the experiments we might do because new things might be discovered in the future that we would like to investigate.

If you agree that we can store your samples for future research, coded samples will be stored at the University of Liverpool and LSTM. These researchers work closely with other scientists in the UK and elsewhere. We would like to allow other researchers, including those

For peer review only - http://bmjopen.bmj.com/site/about/guidelines.xhtml

who also have ideas about coronaviruses, to apply to use your samples for similar work in the future too.

We would like your permission to allow your samples to be transferred outside of the University of Liverpool and LSTM for purposes including those of coronavirus immunity testing. We are asking this now so we don't have to ask you again in the future.

Any future experiments not related to coronaviruses or other common human virus infections would be approved by a research ethics committee. If you don't want your samples stored, that's fine, you can still take part in the rest of the study, we just won't keep your samples at the end. Or if you are happy with having your samples stored, but not sent to another lab, you can choose this as well.

The samples will be kept in a secure place until we need them; nobody outside of the study will have access to **any** confidential information that you give to us. Confidential details (such as your name, address and GP details) will be kept locally and not made available to collaborators.

What if there is a problem?

If you have a concern about any aspect of this study, you should ask to speak with one of the research team who will do their best to answer your questions.

Every care will be taken in the course of this research study. However, in the unlikely event that you are harmed by taking part in this research project of the study Sponsor (University of Liverpool), the Sponsor holds Insurance for the conduct of clinical research. Compensation may be available and you may have to pay your related legal costs.

Thank you for taking the time to read and consider this information sheet. Should you decide to take part in the study, you will be given a copy of the information sheet and a signed consent form to keep.

For peer review only

For peer review only - http://bmjopen.bmj.com/site/about/guidelines.xhtml

COVID-LIV Study

UNIVERSITY OF LIVERPOOL

Liverpool Household COVID-19 Cohort Study (COVID-LIV)

FOR SITE USE ONLY:

Site Name:			
Household ID:		Participant ID	
Participant Initials:		Participant DOB:	/ /
Participant Postcode:	-		

Adult Consent Form

To be completed by the participant:

Once you have read and understood each statement please enter your initials in each box.	Initial
1. I have read and understood the information sheet for this study. I have had the opportunity to ask questions and have had these answered satisfactorily.	
2. I understand that participation is voluntary and that I am free to withdraw from the study at any time, without giving a reason, and without my care or legal rights being affected. However, the study team may need to collect some limited information for safety reasons.	
3. I give permission for a copy of this fully completed consent form to be sent to the LCTC (where it will be kept in a secure location) to allow confirmation that my consent was given.	
4. I understand that relevant sections of my medical notes and any data collected during the study may be looked at by authorised individuals from the central study team and representatives of the Sponsor, and relevant regulatory authorities. I give permission for these individuals to have access to my records and data.	
5. I understand that my data will be kept by the University of Liverpool and all others archiving data in a confidential manner for up to a maximum of 25 years from the end of the study.	
6. I consent to samples of my blood, saliva and stool, and for nose and throat swabs to be taken and used for this study.	
7. I agree to take part in the above study.	
The statements below are optional (you can still take part in the study even if you do not wish to agree to these):	
8. I agree for data previously collected for other research to be looked at by the research team.	
9. I consent to providing an optional stool sample at week 6-8 and 12-14 for the purpose of this study.	
10. I consent to my blood and stool samples being stored by the University of Liverpool for testing for SARS-CoV-2 immunity, and immunity to other coronaviruses.	
11. I agree for samples collected for future research to be stored at The University of Liverpool and LSTM.	
12. I agree for samples collected for future research to be transferred outside of the University of Liverpool and LSTM, along with a copy of this Consent Form, for different tests for coronavirus immunity.	
13. I agree to allow information or results arising from this study to be used in future healthcare and/or medical research providing my confidentiality is maintained.	
14. I agree to my GP being informed of my participation in the study, and if I test positive for COVID-19.	
15. I agree for my GP to be contacted for information relating to my health records, including the provision of my NHS number.	

For peer review only

For peer review only - http://bmjopen.bmj.com/site/about/guidelines.xhtml

COVID-LIV
Study

UNIVERSITY OF LIVERPOOL

Liverpool Household COVID-19 Cohort Study (COVID-LIV)

FOR SITE USE ONLY:

Site Name:														
Household ID:				Participant ID										
Participant Initials:				Participant DOB:			/			/				
Participant Postcode:					-									

Adult Consent Form

16. I agree that I may be contacted in the future in relation to this or other related studies. ☐
If you agree to statement 15 provide your details below:

Telephone number:											
Email address:											

To be completed by the participant:

Your full name (please print):			
Your signature:		Date:	

To be completed by the Researcher (after participant has completed the form):

Researcher full name (please print):			
Researcher signature:		Date:	

Please file the original wet-ink copy in the COVID-LIV Investigator Site File, and make two copies: one for the participant and one to be sent to the LCTC.

For peer review only

For peer review only - http://bmjopen.bmj.com/site/about/guidelines.xhtml

BMJ Open

Prospective observational study of SARS-CoV-2 infection, transmission, and immunity in a cohort of households in Liverpool City Region, UK (COVID-LIV): a study protocol

Journal:	*BMJ Open*
Manuscript ID	bmjopen-2020-048317.R1
Article Type:	Protocol
Date Submitted by the Author:	17-Feb-2021

Complete List of Authors:	Setiabudi, Wega; University of Liverpool Department of Clinical Infection Microbiology and Immunology<br>Hungerford, Daniel; University of Liverpool Department of Clinical Infection Microbiology and Immunology; NIHR Health Protection Research Unit in Emerging and Zoonotic Infections at University of Liverpool<br>Subramaniam, Krishanthi; University of Liverpool Department of Clinical Infection Microbiology and Immunology<br>Vaselli, Natasha; University of Liverpool Department of Clinical Infection Microbiology and Immunology<br>Shaw, Victoria E.; University of Liverpool Institute of Translational Medicine, Liverpool Good Clinical Laboratory Practice<br>Wilton, Moon; University of Liverpool, Department of Psychology<br>Vivancos, Roberto; Public Health England, Field Epidemiology North West, Field Service, National Infection Service; NIHR Health Protection Research Unit in Emerging and Zoonotic Infections at University of Liverpool<br>Aston, Stephen; University of Liverpool Department of Clinical Infection Microbiology and Immunology<br>Platt, Gareth; University of Liverpool Department of Clinical Infection Microbiology and Immunology<br>Moitt, Tracy; University of Liverpool, Liverpool Clinical Trial Centre<br>Jones, Ashley P.; University of Liverpool, Liverpool Clinical Trial Centre<br>Gabbay, Mark; University of Liverpool, Department of Primary Care and Mental Health; NIHR Health Protection Research Unit in Emerging and Zoonotic Infections at University of Liverpool<br>Buchan, Iain; University of Liverpool Department of Public Health and Policy<br>Carrol, Enitan; University of Liverpool Department of Clinical Infection Microbiology and Immunology<br>Iturriza-Gomara, Miren; University of Liverpool Department of Clinical Infection Microbiology and Immunology<br>Solomon, Tom; University of Liverpool Department of Clinical Infection Microbiology and Immunology; NIHR Health Protection Research Unit in Emerging and Zoonotic Infections at University of Liverpool<br>Greenhalf, William; University of Liverpool Institute of Translational Medicine, Liverpool Good Clinical Laboratory Practice<br>Naisbitt, Dean J.; University of Liverpool Department of Molecular and Clinical Pharmacology

	Adams, Emily ; Liverpool School of Tropical Medicine, Department of Tropical Disease Biology; NIHR Health Protection Research Unit in Emerging and Zoonotic Infections at University of Liverpool<br>Cunliffe, Nigel; University of Liverpool Department of Clinical Infection Microbiology and Immunology<br>Turtle, Lance; University of Liverpool Department of Clinical Infection Microbiology and Immunology; NIHR Health Protection Research Unit in Emerging and Zoonotic Infections at University of Liverpool<br>French, Neil; University of Liverpool Department of Clinical Infection Microbiology and Immunology; NIHR Health Protection Research Unit in Emerging and Zoonotic Infections at University of Liverpool
**Primary Subject Heading**:	Epidemiology
Secondary Subject Heading:	Infectious diseases, Immunology (including allergy)
Keywords:	COVID-19, IMMUNOLOGY, Epidemiology < INFECTIOUS DISEASES, Respiratory infections < THORACIC MEDICINE, VIROLOGY

SCHOLARONE™ Manuscripts

I, the Submitting Author has the right to grant and does grant on behalf of all authors of the Work (as defined in the below author licence), an exclusive licence and/or a non-exclusive licence for contributions from authors who are: i) UK Crown employees; ii) where BMJ has agreed a CC-BY licence shall apply, and/or iii) in accordance with the terms applicable for US Federal Government officers or employees acting as part of their official duties; on a worldwide, perpetual, irrevocable, royalty-free basis to BMJ Publishing Group Ltd ("BMJ") its licensees and where the relevant Journal is co-owned by BMJ to the co-owners of the Journal, to publish the Work in this journal and any other BMJ products and to exploit all rights, as set out in our licence.

The Submitting Author accepts and understands that any supply made under these terms is made by BMJ to the Submitting Author unless you are acting as an employee on behalf of your employer or a postgraduate student of an affiliated institution which is paying any applicable article publishing charge ("APC") for Open Access articles. Where the Submitting Author wishes to make the Work available on an Open Access basis (and intends to pay the relevant APC), the terms of reuse of such Open Access shall be governed by a Creative Commons licence – details of these licences and which Creative Commons licence will apply to this Work are set out in our licence referred to above.

Other than as permitted in any relevant BMJ Author's Self Archiving Policies, I confirm this Work has not been accepted for publication elsewhere, is not being considered for publication elsewhere and does not duplicate material already published. I confirm all authors consent to publication of this Work and authorise the granting of this licence.

For peer review only

TITLE

Prospective observational study of SARS-CoV-2 infection, transmission, and immunity in a cohort of households in Liverpool City Region, UK (COVID-LIV): a study protocol

AUTHORS

Wega Setiabudi[1]*, Daniel Hungerford[1,2,3]*, Krishanthi Subramaniam[1], Natasha Marcella Vaselli[1], Victoria E. Shaw[4], Moon Wilton[5], Roberto Vivancos[2,3,6], Stephen Aston[1], Gareth Platt[1], Tracy Moitt[7], Ashley P. Jones[7], Mark Gabbay[3,8], Iain Buchan[2,9], Enitan D. Carrol[1], Miren Iturriza-Gomara[1,2], Tom Solomon[1,3], William Greenhalf[4], Dean J. Naisbitt[10], Emily R. Adams[3,11], Nigel A. Cunliffe[1,2], Lance Turtle[1,3], Neil French[1,3], on behalf of the COVID-LIV Study Group

Affiliations

1. Department of Clinical Infection, Microbiology, and Immunology, Institute of Infection, Veterinary, & Ecological Sciences, University of Liverpool, Liverpool, UK
2. NIHR HPRU in Gastrointestinal Infections at the University of Liverpool, Liverpool, UK
3. NIHR HPRU in Emerging and Zoonotic Infections at the University of Liverpool, Liverpool, UK
4. Liverpool Good Clinical Laboratory Practice, Institute of Translational Medicine, University of Liverpool, Liverpool, UK
5. Department of Psychology, Institute of Population Health, University of Liverpool, Liverpool, UK
6. Field Epidemiology North West, Field Service, National Infection Service, Public Health England, Liverpool, UK
7. Liverpool Clinical Trial Centre, University of Liverpool, Liverpool, UK
8. Department of Primary Care and Mental Health, Institute of Population Health, University of Liverpool, Liverpool, UK
9. Department of Public Health and Policy, Institute of Population Health, University of Liverpool, Liverpool, UK
10. Department of Molecular and Clinical Pharmacology, Institute of Translational Medicine, University of Liverpool, Liverpool, UK
11. Department of Tropical Disease Biology, Liverpool School of Tropical Medicine, Liverpool, UK

Corresponding Author:
Neil French. 8 West Derby Street, Liverpool, L69 7BE, United Kingdom. french@liverpool.ac.uk

*WS and DH contributed equally to this paper.

*WS and DH are joint first authors.

For peer review only

ABSTRACT

Introduction

The emergence and rapid spread of COVID-19 have caused widespread and catastrophic public health and economic impact, requiring governments to restrict societal activity to reduce the spread of the disease. The role of household transmission in the population spread of SARS-CoV-2, and of host immunity in limiting transmission, is poorly understood. This paper describes a protocol for a prospective observational study of a cohort of households in Liverpool City Region, UK, which addresses the transmission of SARS-CoV-2 between household members and how immunological response to the infection changes over time.

Methods and analysis

Households in the Liverpool City Region, in which members have not previously tested positive for SARS-CoV-2 with a nucleic acid amplification test, are followed up for an initial period of 12 weeks. Participants are asked to provide weekly self-throat and nasal swabs and record their activity and presence of symptoms. Incidence of infection and household secondary attack rates of COVID-19 are measured. Transmission of SARS-CoV-2 will be investigated against a range of demographic and behavioural variables. Blood and faecal samples are collected at several time points to evaluate immune responses to SARS-CoV-2 infection and prevalence and risk factors for faecal shedding of SARS-CoV-2, respectively.

Ethics and dissemination

The study has received approval from the NHS Research Ethics Committee; REC Reference: 20/HRA/2297, IRAS Number: 283464. Results will be disseminated through scientific conferences and peer-reviewed open access publications. A report of the findings will also be shared with participants. The study will quantify the scale and determinants of household transmission of SARS-CoV-2. Additionally, immunological responses before and during the different stages of infection will be analysed, adding to the understanding of the range of immunological response by infection severity.

STRENGTHS AND LIMITATIONS OF THIS STUDY

- COVID-LIV is a prospective cohort study of households that aims to represent the socioeconomic profile of Liverpool City Region population, which enables the determination of risk factors of SARS-CoV-2 transmission whilst minimising recall bias.
- This household-based study will identify paucisymptomatic and asymptomatic COVID-19 cases, thus allowing the measurement of their contribution to transmission.
- The longitudinal nature of the study enables the capture of subjects before they test positive for COVID-19, which provides a pre- and post-infection time point to evaluate changes to the host immune response.
- Limitations include the relatively small sample size and repeated self-sampling, which may lead to diagnostic inconsistencies.
- Participation bias by those most engaged with COVID-19 and disease control may theoretically result in an unrepresentative study cohort.

For peer review only

INTRODUCTION

Within months of the first reports of a novel respiratory disease in Wuhan, China in December 2019, COVID-19 has been declared a global pandemic with devastating impacts.[1] The disease caused by the severe acute respiratory syndrome coronavirus 2 (SARS-CoV-2) has reached 46.8 million cases and 1.2 million deaths globally as of 3 November 2020, although the real number is likely to be much higher.[2] With limited evidence of effective prophylactic treatment and prior to widespread availability of vaccination, countries around the world have been forced to implement various forms of restriction to individual movement in order to control the transmission of SARS-CoV-2.[3–5] Although the effectiveness of such measures in controlling viral transmission comes at the cost of disruption in socioeconomic activity and mental wellbeing, the impact from uncontrolled transmission could cause the loss of millions of lives and the potential collapse of health systems.[6–9]

Understanding the pattern of community transmission is essential to inform approaches to contain the spread of SARS-CoV-2. The role of transmission between household members is believed to have a significant role in the spread of the disease, where the secondary attack rate is estimated to be 16.6%.[10] This is reflected in the current UK government guideline where members of the household of a confirmed case are required to self-isolate for ten days.[11] Despite this, the limited availability of long-term prospective cohort studies means that further exploration of how SARS-CoV-2 transmits within households, and a better understanding of how immune responses develop over time, are urgently needed.[10,12]

In October 2020, the Liverpool City Region became the first area in England, UK to be placed in the highest level of regional restriction after experiencing one of the highest rate of infection in the country.[13] The region was also chosen as the site for the pilot asymptomatic mass testing due to its high infection rate during the second national lockdown.[14] The transmission characteristic of the Liverpool City Region could be explored further through a community study of the virus transmission. These data would aid understanding of the transmission dynamics of SARS-CoV-2 that may be beneficial in informing public health interventions.

The Liverpool Household COVID-19 Cohort Study (COVID-LIV) is a prospective observational study of households in the Liverpool City Region. As a household-based study, COVID-LIV will capture paucisymptomatic and asymptomatic COVID-19 cases. This allows measurement of the role of different disease manifestation of COVID-19 cases in the transmission of SARS-CoV-2 between household members. In addition, the prospective nature of the study allows characterisation of the immune response to SARS-CoV-2 at different stages of the infection and determine the durability of the response.

STUDY AIMS

Among households in the Liverpool City Region, the primary aim is to understand household associated SARS-CoV-2 transmission. This aim will be achieved by:

1. Measurement of household COVID-19 incidence and secondary attack rates
2. Identification of the determinants of transmission of SARS-CoV-2
3. Estimation of the contribution of paucisymptomatic and asymptomatic infection to the spread of SARS-CoV-2

For peer review only

Secondary aims are:

1. Measure family member contact patterns and the relationship to household structure
2. Describe the clinical phenotype of mild COVID-19 cases
3. Undertake sequence SARS-CoV-2
4. Characterise the immune response in mild COVID-19 cases
5. Characterise the immune response of exposed household contacts with no subsequent detection of confirmed infection
6. Investigate the prevalence of household faecal shedding of SARS-CoV-2

METHODS AND ANALYSIS

Design

COVID-LIV is a prospective observational cohort study of households in the Liverpool City Region, UK. Cohorts are followed up for an initial period of 12 weeks and then up to three years, observing the incidence of household transmission of SARS-CoV-2 and characterising changes in the immune response over time. For 12 weeks, all household members are requested to perform weekly self-administered throat and nasal swabbing, along with the collection of blood and other clinical samples at different time points of the study. The study started in July 2020 and is expected to continue until September 2023.

There are social science studies linked to this household study, including longitudinal surveys of all these households focusing on the impacts of the pandemic on the residents included in this research. In addition, there will be in-depth qualitative interviews at baseline and three months with a purposive sample of these households, focusing on risk perception beliefs and actions.

Study population

Households are recruited from the large metropolitan Liverpool City Region in North West England, UK. The Liverpool City Region comprises six local authorities and has a population of over 1.5 million people. Almost 50% of its population are categorised as living in the 20% most deprived areas of England.[15]

Recruitment procedure

Households were recruited from the established Liverpool household survey undertaken by the NIHR Collaboration for Leadership in Applied Health Research and Care (CLAHRC, now ARC).[16] The established study framework contains over 7000 households, representing a spectrum of demographic and socioeconomic characteristics. The initial selection process was undertaken by ARC team members with appropriate permission to access the original survey data. Individuals who indicated a willingness to be re-contacted for further research participation were identified and contacted by the COVID-LIV study team about their potential participation in this study.

To supplement the number of recruits, other methods to reach out to potential participants were utilised, which include text messages sent from local general practitioner (GP) surgeries to their patients containing information about the study; and sharing study information through the University of Liverpool media, local media outlets, and the social media of the researchers and stakeholder organisations. Interested households that contacted the study team are recruited if they fulfil the study criteria as follows:

For peer review only

Inclusion criteria

1. All people in the household willing to take part
2. At least one adult within the household must speak English and willing to translate
3. Have provided informed verbal and written consent, or personal legal consent for those lacking capacity
4. Ability and willingness to undertake self-swabbing
5. Intention to be resident for at least six months within their current household – except for students, military personnel, and other professions who may have to move away from home for purposes of study or employment

Exclusion criteria

1. Contraindication to throat and nasal swab or blood sampling
2. One or more members of the household have had a proven COVID-19 test (positive PCR test for SARS CoV-2)
3. No members of the household can speak English

A household comprises those individuals who reside at the household at the date of contact, even if they do not believe this is their primary residence and are intending to stay for at least six months beyond the date of enrolment and first sampling. Regularly attending persons such as carer and cleaner are classified as attendees and are asked to participate in the study, although their participation status does not affect the eligibility of the household. A participant aged 16 years or above is considered an adult.

Participant pathway

This section provides the pathway details of study participant from enrolment up to the end of the study (Figure 1).

Consent procedure

The consent process consists of two phases; an introductory communication, followed by a visit to establish consent and sampling. At the first phase, potential households from the list of contacts from ARC will be contacted by phone or email wherever possible for ease and speed of communication and to minimise transmission risk. The same method of communication applies to participants that directly contacted the study team in response to advertisement through GP surgeries and other forms of media communication. During this phase, potential participants are given a brief explanation about the study, access to information on the study website is confirmed, and any queries are answered.

Following initial contact and expression of interest by the household, a visit arrangement by research nurses is made. During the visit, where every household member is expected to be present, printed information sheets are provided along with further discussions on the purpose of the study and procedures required (see online supplementary appendix file 1). Written informed consent is expected to be provided for each member of the household. In addition to the parent or guardian consent form, children are provided with age-relevant information sheets; assent is obtained if the child is aged 8 - 15 years old and deemed capable of assessing the study documentation provided.

Baseline visit

After written consent forms have been acquired from all household members, a baseline visit date is arranged. The visit is done on the day the consent forms are signed, or another date is arranged if necessary. During the baseline visit, the research nurses collect throat and nasal swab, blood samples (or finger prick sample if not suitable for venepuncture), nasal mucosal sample, and saliva sample from

For peer review only

all adult participants. Only a finger prick sample and saliva sample are collected from children. The research nurses also train the participants on how to perform throat and nasal swabbing themselves. Instruction on the procedure of self-swabbing is given to each household, along with the swab kits for the following weeks.

The first 12 weeks of participation

Participants are instructed to perform self-throat and nasal swab every week for a total of 12 weeks after enrolment; samples are collected by a courier. A questionnaire is sent each week through email or phone call if no email address is provided, requesting information about the participant's health condition and activities from the past seven days. Participants are also asked to report any COVID-19 test done outside the study system, both during and after the initial 12 weeks of self-swabbing. Optional stool samples are collected from consenting participants at weeks 6-8 and 12-14 from enrolment.

Positive swab and result notification

If a positive SARS-CoV-2 swab result occurs during the first 12 weeks of self- throat and nasal swabbing, participants are informed of the result within 72 hours of sample receipt at the laboratory. Positive case details are passed to the National Health Service (NHS) test and trace according to Public Health England statutory requirement. Upon notification, participants are given information on self-isolation and are provided with other relevant guidelines from the UK government and the NHS. The participant's GP is also informed, and additional clinical advice is available from the infectious diseases team at Liverpool University Hospitals NHS Foundation Trust or Alder Hey Children's Hospital NHS Foundation Trust if deemed necessary by the study clinical team.

Following notification, a household visit is arranged to obtain additional samples of blood (or finger prick), throat and nasal swab, nasal mucosal swab, and saliva from the positive case and other household members. The visit is expected to be done within three days following result notification; repeat visits are arranged at 14 days and 12 weeks after result notification. If a participant has more than one positive PCR swab result during the course of the study, this will trigger re-start of the additional sampling schedule if more than six weeks have elapsed from the first positive test in order to identify re-infection.

Follow-up visits

The first follow-up visit is performed at 12 weeks after enrolment for households with and without COVID-19 cases. Samples of blood (or finger prick) are collected from households with no positive case, whilst samples of blood (or finger prick), throat and nasal swab, nasal mucosal swab, and saliva are collected from households with at least one positive case. Repeat visits to households with a history of a positive throat and nasal swab, or seropositivity at baseline or at 12-week follow-up, will be conducted at six months, one year, two years, and three years post enrolment.

For peer review only

Clinical sample and laboratory investigation

The following section provides more detail on the clinical samples that are obtained at different time points during the study (Table 1).

Table 1. Collection of clinical samples for the COVID-LIV study

**Timepoint**	**Baseline / enrolment**	**Week 1 - 12 (Day 8, Day 15 etc.)**	**Weeks 6-8 and 12-14**	**Any time in weeks 0-12**	**Week 12 follow-up**	**Week 12, Month 6, Year 1,2, 3**
Participant group	All participants	All participants	All participants[2]	PCR swab positive participants/household members of swab positive participants[3]	PCR swab negative participants	Seropositive / PCR swab positive participants and their household members
Throat & Nasal Swab	X	X				
Blood samples	X			X	X	X
Stool Sample			X[2]	X		
Nasal mucosal sample	X[1]			X[1]		X[1]
Saliva sample	X			X		X

[1] Adult participant only
[2] Optional - additional consent required
[3] Samples taken at day three and day 14 after PCR swab positive test

Throat and nasal swab

A combined throat and nasal swab are taken for detection of SARS-CoV-2 at baseline by the participant under the guidance from the research nurses. Swabs are then taken by the participants at home and collected on a weekly basis for 12 weeks. This sample is collected using nylon flocked dry swabs placed into plastic tubes and transported to the laboratory to be tested within 72 hours. Participants are asked to perform self-swabbing on the night before or in the morning of collection day, where samples are then collected within 12 to 24 hours. Samples are placed inside a specimen bag and specimen cardboard box and stored at ambient temperature until collection and during transport. Swabs are processed for RNA extraction (Zymo Research) and qPCR (Primer Design Novacyt). Remaining amies medium and extracted nucleic acid will be stored for future virology research.

Virus sequencing

Nucleic acid from positive throat and nasal swabs (and a small number of negative swabs as controls) will be transferred to the Centre for Genomic Research, University of Liverpool for SARS-CoV-2 whole-genome sequencing using the nanopore technology and the ARTIC network protocol.[17,18]

For peer review only

Blood sample

Up to 60 ml of whole blood are collected from each adult participant (or finger prick sample, if unsuitable for venepuncture). Children under the age of 16 years will have finger prick and blood spot collection rather than venepuncture, and a smaller amount of blood collected.

The baseline and 12-week follow-up blood samples will be used to determine the prevalence of exposure to SARS-CoV-2 at a certain point of the epidemic. These data will be used to supplement virology data to maximise the identification of SARS-CoV-2 exposure.

Peripheral blood mononuclear cells (PBMCs) will be isolated from different time points of infection using Ficoll density centrifugation. Briefly, blood collected from sodium heparin tubes will be placed on a Ficoll cushion and centrifuged to retrieve PBMCs. Cells will then be washed and frozen down in 90% fetal bovine serum (FBS) and 10% DMSO for downstream assays.

Antibody responses

The antibody response will be measured over time at baseline, 12 weeks, 6 months, and 1-, 2- and 3-years post-infection by ELISA, pseudo-virus neutralisation and SARS-CoV-2 neutralisation in a subset. The proportion of participants positive at each time point will be determined, and the magnitude of antibody titres measured. If positive cases are detected, neutralising antibody titres will be measured, and virus isolation will be attempted allowing testing of the serum neutralising capacity against the actual virus infecting the participant. Mucosal antibody and cytokine responses will be tested. These experiments will determine whether serum antibody measurements correlate with mucosal antibody and whether either is an adequate correlate of immunity. Parallel samples from household contacts (who are highly likely to be exposed) will also be collected and studied in order to determine what, if any, factors protect against the acquisition of infection, or correlate with sterilising immunity.

T cell responses

Antigen-specific responses will be measured following *ex-vivo* stimulation with SARS-CoV-2 peptide pools. PBMCs isolated at the various time points will be stimulated with various peptide pools and ICS (intracellular cytokine stain) and activation marker assays will be performed to characterise the SARS-CoV-2 T cell responses. Single-cell RNA-seq assays will also be done to explore the breadth of the T cell response to determine qualitative differences in the T cell repertoire. Where sample allows, T cell epitopes will be mapped using a synthetic peptide library and tested for cross-reactivity against common cold coronaviruses.

Innate response

Whole blood stored in RNA stabilisation solution (tempus tubes) will be subjected to RNA isolation and sequencing to characterise the innate immune response. These data will inform and refine the above experiments and have the potential to be related to the ISARIC 4C dataset (hospitalised severe cases) as a mild disease group.[19]

Genomic testing

Human leukocyte antigen typing will be undertaken along with characterisation of other important immune mediating characteristics, such as Angiotensin reception 2 (ACR2).

Stool sample

Stool samples are collected from adult participants who test positive from a PCR swab and from their consenting household contacts at approximately 3 and 14 days after confirmation of a positive PCR. Samples are transported to the University of Liverpool where they are frozen down for downstream

For peer review only

assays, including for SARS-CoV-2 sequencing. Additionally, optional stool samples are requested from all participants at two time points from their enrolment at approximately week 6 – 8 and week 12 – 14.

Nasal mucosal and saliva sample

At the baseline visit, nasal mucosal and saliva samples are collected from adult participants and all participants, including children, respectively. The nasal mucosal sample is collected using synthetic absorptive matrix (SAM) strips, and saliva sample is collected using ORACOL+ (Malvern Medical Developments) , both are collected for antibody analyses. Additional samples are also collected from adult participants who subsequently tested positive from PCR swab and their household contacts.

Outcome measures

Primary endpoints

1. Incidence of paucisymptomatic and asymptomatic SARS-CoV-2 infections index cases, including the prevalence of infection or past infection at baseline serology status
2. Incidence of secondary household cases
3. Risk factors for household transmission

Secondary endpoints

1. Analysis of household contact patterns
2. Description of clinical phenotypes of the index cases
3. Genomic characterisation of SARS-CoV-2
4. Characterisation of the immune response in index cases and exposed household contacts
5. Prevalence of SARS-CoV-2 household faecal shedding

Data analysis

The results of the analyses will be reported according to the STROBE guidelines.[20] This will include a descriptive analysis of households, paucisymptomatic and asymptomatic primary household index cases, and secondary household cases.

Environmental, demographic, and behavioural risk factors for secondary transmission among household contacts of symptomatic and laboratory-confirmed cases of COVID-19 will be investigated. Cases in households will be ordered by date of symptom onset. The first symptomatic COVID-19 case in the household will be classified as the probable household index case. Secondary COVID-19 cases will be defined as any COVID-19 case with an onset of illness within seven days following the onset of the index case.

The primary attack rate will be calculated as the number of households with a primary case divided by the total number of households in the study. The household secondary attack rate will be calculated from the number of households with at least one secondary COVID-19 case divided by the total number of households at risk. The individual household members attack rate will be calculated from the number of household members with secondary COVID-19 illness divided by the total number of household members at risk.

Index cases will be described in relation to demographics, employment, and contact history. A risk factor analysis will be undertaken to investigate variables associated with secondary attack cases within households. Risk factors will include data on contacts, viral load measurements, household characteristics, and other variables that emerge in external reports or literature that may be linked with transmission.

For peer review only

Data and statistical analysis of serology and other immunological parameters will be done using GraphPad Prism, FlowJo v10, R and other bioinformatics software.

Sample size

The study should be regarded as exploratory. The initial constraints on sample size are primarily access to testing on a weekly basis. Referring to the current data on the secondary attack rate (SAR) of 10.5 – 45% of contacts with a hazard ratio of 1.5 or 2.0, using a single sample Cox proportional hazard model with 80% power and 10% study withdrawal, we propose an initial sample size of 300 households, which will contain approximately 1000 individuals.[21–23]

Patient and public involvement

The protocol has been reviewed by the patient and public involvement (PPI) committee of the Institute of Infection, Veterinary, & Ecological Sciences, University of Liverpool. The study design, participant acceptability, and perceptions have been reviewed and discussed. The necessary speed to get this work up and underway has prevented a more standard input from the PPI group. Test results will be reported to participants in plain language.

ETHICS AND DISSEMINATION

The study has received approval from the NHS Research Ethics Committee; REC Reference: 20/HRA/2297, IRAS Number: 283464. Protocol amendments have been and will be reported to the Research Ethics Committee, as will any serious adverse events. The study participants are informed that all data collected are for research purposes only and that they have the right to withdraw from the study at any time.

PROJECT GOVERNANCE

The study is coordinated by the Liverpool Clinical Trials Centre, University of Liverpool. A study steering group has been established to enable effective achievement of the project objectives. The steering group includes representatives from academia, public health, and lay membership.

DISSEMINATION OF RESEARCH FINDINGS

The findings will be presented at professional and scientific conferences. The results will also be published in peer-review publications and if appropriate, published first as pre-prints to enable a timely public health response to COVID-19. Interim and final reports will be submitted to the funders and the steering group. We also work with our institute PPI panel to identify and produce materials to disseminate to the general public, including study participants.

DISCUSSION

COVID-LIV will demonstrate the role of household transmission of SARS-CoV-2 in a cohort of households in the Liverpool City Region, UK. By observing households with no apparent previous infection of COVID-19, it is hoped that the incidence, determinants of transmission, and contribution of paucisymptomatic and asymptomatic cases can be described, filling a knowledge gap in how the disease transmits within the population in the Liverpool City Region. Characterising immune responses in a cohort of mild infections will provide a better understanding of how natural infection alters immune parameters over time, allowing a better understanding of immunity against COVID-19 infection that may help inform vaccine development and delivery.

Strengths

COVID-LIV aimed to recruit a cohort of households across a representative range of socioeconomic status in the Liverpool City Region. The household cohort allows for identification of paucisymptomatic and asymptomatic COVID-19 cases, which will provide a better representation of the impact of COVID-19 in the community and extent of transmission through the sampling of high probability exposed household members. The prospective nature of the study allows the determination of a true incidence rate and risk factors for SARS-CoV-2 transmission with less recall bias. The longitudinal study design enables the analysis of the immunological response and faecal shedding of SARS-CoV-2 during different stages of the disease. It also allows observation of the natural progression of mild cases from a pre-infection stage sample collection to allow the interrogation of T cell repertoires and their association with acute infection.

Limitations

The cohort households may be biased by those that are most engaged with COVID-19 and disease control, leading to a low level of secondary infections as participating individuals are more likely to take precautions against transmission. Low level of detectable infections may also be observed due to the study observation across different seasonality time points. Reliance on repeated self-sampling may lead to diagnostic inconsistencies, although instructions were given, and techniques were carefully assessed by the research nurses during the initial baseline visit. Exclusion of non-English speaking families may exclude potential high-risk households resulting in under detection of incidence rate and more severe cases.

For peer review only

ACKNOWLEDGEMENT

The authors would like to thank and appreciate all the participants in the study for their invaluable contribution and the external advisory panel members: Jonathan M. Read, Antonia Ho, and Cliona McDowell.

The following are members of the COVID-LIV Study Group:

Principal investigator: Neil French

Study Investigators: Lance Turtle, Daniel Hungerford, Krishanthi Subramaniam, Roberto Vivancos, Mark Gabbay, Iain Buchan, Enitan D. Carrol, Miren Iturriza-Gomara, Tom Solomon, Nigel A. Cunliffe, Emily R. Adams, Carrol Gamble

Lay members: Lynnette Crossley, Neil Joseph

Fieldwork team: Wega Setiabudi, Natasha Marcella Vaselli, Moon Wilton, Lee D. Troughton, Samantha Kilada, Katharine Abba, Victoria Simpson, John S.P. Tulloch, Lynsey Goodwin, Rachael Daws, Shiva Seyed Forootan, Susan Dobson, Rachel Press, Vida Spaine, Lesley Hands, Kate Bradfield, Carol McNally

Project management: Tracy Moitt, Silviya Balabanova, Chloe Donohue, Lynsey Finnetty, Laura Marsh

Clinical and laboratory team: William Greenhalf, Dean J. Naisbitt, Victoria E. Shaw, Stephen Aston, Gareth Platt, Paul J. Thomson, Monday Ogese, Sean Hammond, Kareena Adair, Liam Farrell, Joshua Gardner, Kanoot Jaruthamsophon, Serat-E Ali, Adam Lister, Laura Booth, Milton Ashworth, Katie Bullock, Benjamin W.A. Catterall, Terry Foster, Lara Lavelle-Langham, Joanna Middleton, William Reynolds, Emily Cass, Alejandra Doce Carracedo, Lianne Davies, Lisa Flaherty, Melanie Oates, Nicole Maziere, Jennifer Lloyd, Christopher Jones, Hannah Massey, Anthony Holmes, Nicola Carlucci, Vanessa Brammah, Yasmyn Ramos, Daniel Allen, Jane Armstrong, Debbie Howarth, Eve Wilcock, Jena Lowe, Jayne Jones, Paula Wright, Iain Slack, Simone McLaughlin, Jessica Mason, Thomas Edwards, Claudia McKeown, Elysse Hendrick, Chris Williams, Rachel Byrne, Kate Buist, Gala Garrod, Sophie Owen

Statisticians: Ashley P. Jones, Efstathia Gkioni

AUTHOR'S CONTRIBUTION

NF, NAC, DH, LT, MI-G conceived of the study. DH, KS, NF, NAC, LT, MI-G, TS, SA, IB, MG, RV, MW, NMV, WS, EDC, ERA initiated study design and protocol development. GP, VES, WG, DJN, TM helped with study implementation. DH, NF, APJ provide statistical expertise in statistical design and have produced the analysis plan. WS and DH drafted the manuscript. All authors contributed to the refinement of the study protocol.

FUNDING STATEMENT

This study is co-funded by the National Institute for Health Research Health Protection Research Unit (NIHR HPRU) in Gastrointestinal Infections, a partnership between Public Health England, the University of Liverpool and the University of Warwick; the NIHR HPRU in Emerging and Zoonotic Infections, a partnership between Public Health England, the University of Liverpool in collaboration with the Liverpool School of Tropical Medicine and the University of Oxford; the Centre of Excellence in Infectious Disease Research (CEIDR); and the Alder Hey Charity. Grant number for the fundings is not applicable.

NF is funded by the National Institute for Health Research Health Protection Research Unit (NIHR HPRU) in Emerging and Zoonotic Infections, the Centre of Excellence in Infectious Diseases Research (CEIDR), and the Alder Hey Charity. We also acknowledge the support of Liverpool Health Partners and the Liverpool-Malawi-Covid-19 Consortium.

This research was funded in whole, or in part, by a Wellcome Trust fellowship awarded to LT (205228/Z/16/Z). For the purpose of Open Access, the author has applied a CC BY public copyright licence to any Author Accepted Manuscript version arising from this submission. LT is also supported by the National Institute for Health Research Health Protection Research Unit (NIHR HPRU) in Emerging and Zoonotic Infections (NIHR200907) at University of Liverpool in partnership with Public Health England (PHE), in collaboration with Liverpool School of Tropical Medicine and the University of Oxford. LT is based at University of Liverpool.

For peer review only

WS is funded by the Ministry of Finance, the Republic of Indonesia through the Indonesia Endowment Fund for Education (Lembaga Pengelola Dana Pendidikan or LPDP) scholarship for doctoral study (201807220413052).

DH is funded by a National Institute for Health Research (NIHR) Post-doctoral Fellowship (PDF-2018-11-ST2-006).

KS is funded by a HEFCE-funded University of Liverpool Tenure Track Fellowship.

EDC acknowledges funding from the NIHR i4i Programme (II-LA-0216-20002), HTA Programme (15/188/42, 17/136/13), EME Programme (NIHR129960) and H2020 (Project No. 848196).

MG is part-funded by the NIHR Applied Research Collaboration North West Coast.

The views expressed are those of the author(s) and not necessarily those of the NHS, the NIHR, the Department of Health and Social Care, Public Health England, or other funding bodies.

COMPETING INTERESTS STATEMENTS

MW is funded under a grant from Astra Zeneca and University of Liverpool for an unrelated project.

REFERENCES

1. Walker PG, Whittaker C, Watson O, et al. The Global Impact of COVID-19 and Strategies for Mitigation and Suppression. *Imperial College COVID-19 Response Team*. 2020;March(June):19. doi.org/10.25561/77735.

2. Coronavirus disease (COVID-19). https://www.who.int/emergencies/diseases/novel-coronavirus-2019. Accessed November 3, 2020.

3. Boulware DR, Pullen MF, Bangdiwala AS, et al. A Randomized Trial of Hydroxychloroquine as Postexposure Prophylaxis for Covid-19. *New England Journal of Medicine*. 2020;383(6):517-525. doi:10.1056/nejmoa2016638

4. UK medicines regulator gives approval for first UK COVID-19 vaccine - GOV.UK. https://www.gov.uk/government/news/uk-medicines-regulator-gives-approval-for-first-uk-covid-19-vaccine. Accessed December 16, 2020.

5. Coronavirus: The world in lockdown in maps and charts - BBC News. https://www.bbc.co.uk/news/world-52103747. Accessed November 3, 2020.

6. Flaxman S, Mishra S, Gandy A, et al. Estimating the effects of non-pharmaceutical interventions on COVID-19 in Europe. *Nature*. 2020;584(7820):257-261. doi:10.1038/s41586-020-2405-7

7. The Global Economic Outlook During the COVID-19 Pandemic: A Changed World. https://www.worldbank.org/en/news/feature/2020/06/08/the-global-economic-outlook-during-the-covid-19-pandemic-a-changed-world. Accessed November 3, 2020.

For peer review only

8. McGinty EE, Presskreischer R, Han H, Barry CL. Psychological Distress and Loneliness Reported by US Adults in 2018 and April 2020. *JAMA - Journal of the American Medical Association*. 2020;324(1):93-94. doi:10.1001/jama.2020.9740

9. Sachs JD, Abdool Karim S, Aknin L, et al. Lancet COVID-19 Commission Statement on the occasion of the 75th session of the UN General Assembly. *The Lancet*. 2020;396(10257):1102-1124. doi:10.1016/S0140-6736(20)31927-9

10. Madewell ZJ, Yang Y, Longini IM, Halloran ME, Dean NE. Household Transmission of SARS-CoV-2: A Systematic Review and Meta-analysis. *JAMA Network Open*. 2020;3(12):e2031756. doi:10.1001/jamanetworkopen.2020.31756

11. Stay at home: guidance for households with possible or confirmed coronavirus (COVID-19) infection - GOV.UK. https://www.gov.uk/government/publications/covid-19-stay-at-home-guidance/stay-at-home-guidance-for-households-with-possible-coronavirus-covid-19-infection. Accessed December 15, 2020.

12. Koh WC, Naing L, Chaw L, et al. What do we know about SARS-CoV-2 transmission? A systematic review and meta-analysis of the secondary attack rate and associated risk factors. Leekha S, ed. *PLOS ONE*. 2020;15(10):e0240205. doi:10.1371/journal.pone.0240205

13. Liverpool City Region to move into "very high" local COVID Alert Level following rise in coronavirus infections - GOV.UK. https://www.gov.uk/government/news/liverpool-city-region-to-move-into-very-high-local-covid-alert-level-following-rise-in-coronavirus-infections. Accessed November 5, 2020.

14. Liverpool to be regularly tested for coronavirus in first whole city testing pilot - GOV.UK. https://www.gov.uk/government/news/liverpool-to-be-regularly-tested-for-coronavirus-in-first-whole-city-testing-pilot. Accessed December 16, 2020.

15. Liverpool City Council. The Index of Multiple Deprivation 2019 A Liverpool analysis Executive Summary. 2019. https://liverpool.gov.uk/media/1359213/imd-2019-liverpool-analysis-main-report.pdf.

16. Giebel C, McIntyre JC, Daras K, et al. What are the social predictors of accident and emergency attendance in disadvantaged neighbourhoods? Results from a cross-sectional household health survey in the north west of England. *BMJ Open*. 2019;9(1):e022820. doi:10.1136/bmjopen-2018-022820

17. Quick J. nCoV-2019 sequencing protocol. *protocols.io*. 2020.

18. Fernández-Rodríguez A, Casas I, Culebras E, Morilla E, Cohen MC, Alberola J. COVID-19 and post-mortem microbiological studies. *Spanish Journal of Legal Medicine*. 2020;46(3):127-138. doi:10.1016/j.remle.2020.05.007

19. ISARIC4C consortium. https://isaric4c.net/. Accessed February 12, 2021.

20. von Elm E, Altman DG, Egger M, Pocock SJ, Gøtzsche PC, Vandenbroucke JP. The Strengthening the Reporting of Observational Studies in Epidemiology (STROBE) Statement: Guidelines for Reporting Observational Studies. *PLoS Medicine*. 2007;4(10):e296. doi:10.1371/journal.pmed.0040296

21. Liu Y, Eggo RM, Kucharski AJ. Secondary attack rate and superspreading events for SARS-CoV-

For peer review only

2. *The Lancet*. 2020;395(10227):e47. doi:10.1016/S0140-6736(20)30462-1

22. Burke RM, Midgley CM, Dratch A, et al. Active Monitoring of Persons Exposed to Patients with Confirmed COVID-19 — United States, January–February 2020. *MMWR Morbidity and Mortality Weekly Report*. 2020;69(9):245-246. doi:10.15585/mmwr.mm6909e1

23. Bi Q, Wu Y, Mei S, et al. Epidemiology and transmission of COVID-19 in 391 cases and 1286 of their close contacts in Shenzhen, China: a retrospective cohort study. *The Lancet Infectious Diseases*. 2020;20(8):911-919. doi:10.1016/S1473-3099(20)30287-5

FIGURE LEGENDS

Figure 1: Participant pathway

For peer review only

Figure 1. Participant pathway

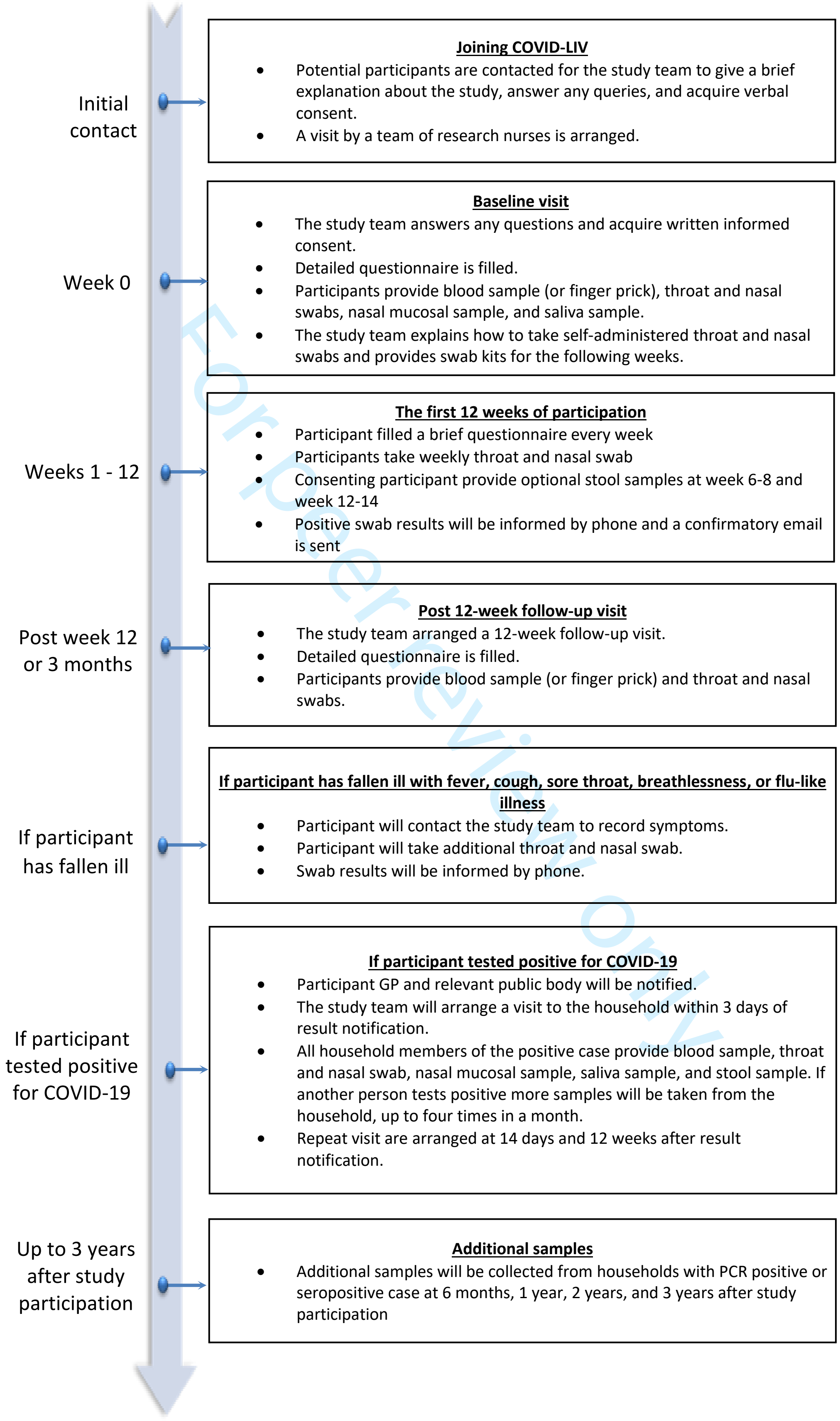

For peer review only

Liverpool Household COVID-19 Cohort Study (COVID-LIV)

Adult Information Sheet for COVID-LIV

- You were contacted by telephone or you have contacted us and verbally agreed to take part in a research study called COVID-LIV. You also provided information about yourself.
- Please now take time to read the following information carefully (note, you may have received this information via email following the telephone call). **Part 1** tells you the purpose of the study and what will happen to you if you take part. **Part 2** gives you more detailed information about the conduct of the study.
- You can ask a member of the research team if there is anything that is not clear, or if you would like more information.
- Taking part is voluntary. If you don't want to take part then you don't need to give a reason.
- If you want to take part but other members of the household do not want to, we may not be able to include you in the study.
- The COVID-LIV study team are studying how COVID-19 spreads in the community, inside households.
- This is being done using swab tests – the same tests that you would have if you came into hospital with COVID-19. If you choose to, you can also provide a stool sample at 6-8 and 12-14 weeks.
- We need all kinds of different households across Liverpool city region to take part.
- The study is currently funded for 12 months whilst additional funding is obtained to continue for a further three years.

How to contact the study team:

If you have any questions about this study, please talk to a member of the study team who visits you, or call: ###

Professor Neil French is the lead Investigator.

Contents:

Part 1 – Purpose of the study and what will happen if you take part

- Why are we doing the COVID-LIV study?
- Why have I been chosen?
- Do I have to take part?
- What will happen to me if I take part?
- What will I have to do if I take part?
- What are the benefits and risks of taking part?
- What happens if I change my mind?
- What happens when the study stops?
- What if there is a problem?
- Will my taking part in the study be kept confidential?

Part 2 – Detailed information about the conduct of the study

- Who is running the study?
- How will my information be collected and handled?
- What are my choices about how my information is used?
- Information sharing for other research
- Where can I find out more about how my information is used?
- What will happen to the nose and throat swabs, and the blood, saliva and stool samples I give?
- What if there is a problem?

If you become ill or suspect you have COVID-19 please follow government guidelines

For peer review only - http://bmjopen.bmj.com/site/about/guidelines.xhtml

PART 1: Purpose of the study and what will happen if you take part

Why are we doing the COVID-LIV study?

COVID-19 (or COVID for short) is the name of the disease that is caused by the new coronavirus. This coronavirus was first found in China in January 2020. The new coronavirus has since spread all round the world. COVID-19 is caused by a virus strain called SARS-CoV-2. We want to understand what factors determine transmission of the virus and how our bodies respond and become resistant to it.

Most people who get COVID-19 will be fine as it is not a serious disease in most people. Some people might not even know that they had it. But in a few people, COVID-19 makes them very sick. They may need a ventilator to help them breathe, or may even die. In order to prevent this, the government has asked everyone except essential workers to stay at home whenever possible. Whilst this will work, it has other effects, for example preventing people from working, so there is a great cost to the country, and to our personal freedoms. We need to understand how COVID-19 spreads, so we can help tell the government when to stop advising people to stay home, and what might happen as they do tell us we can go out again.

The results from this study will be used to provide valuable information for the government and local public health to plan the next stage of the COVID-19 response – that is how we step down from the lock down and back to more contacts and interactions.

Why have I been chosen?

You, and your household, have either been selected because you were part of another study before, called CLAHRC NWC Household Health Survey or you have responded to one of our communications seeking volunteers. Those in the household study gave permission to be re-contacted again about other studies. Therefore, we are contacting you to ask if you would like to take part in this study.

We are selecting different types of households (for example with different numbers of people, or those with and without children) to take part in COVID-LIV. We need all different kinds to take part so we have not approached you based on anything particular about you or your household, or family. We are looking for around 1000 people from 300 different households to take part, so that we can be sure the people in the study are just like those in the whole community.

Do I have to take part?

No, taking part is voluntary. It is up to you to decide whether or not you want to take part.

We also want all the members of the household to take part. If you want to but other members of the household do not want to participate we may not be able to include you.

If you do decide to take part, we will ask you to sign a consent form.

If you decide to take part, you can also choose to stop at any time without giving a reason.

The decision you make on whether to take part or not will not affect the care you receive for COVID-19 now, or in the future.

What will happen to me if I take part?

After you have signed the consent form, we will ask you to complete a questionnaire (we will ask you about members of your household, diet, employment, and some other things). The researcher will take up to 60ml blood sample from you. This sounds like a lot, but it is in fact only about 1½ egg cups in size. For the participants unable to donate blood, the researcher will take a finger prick test and blood spot collection. We will also collect a sample of saliva with a mini sponge and a sample of nasal fluid with a small piece of special filter paper. We will then explain how you can take nose swabs from yourself or other members of the household and ask you to do the first swab in the presence of a researcher to check you are able to do it correctly. These swabs are for COVID-19 testing, and are easy to use. If you feel ill at the time of the first visit we will only take the nose swab to see if you have COVID19 and arrange a further visit to collect blood and other samples depending on the swab result. You will swab your nose once a week, for 12 weeks (or at any other time if you think you have fallen

For peer review only

For peer review only - http://bmjopen.bmj.com/site/about/guidelines.xhtml

ill with fever, cough, sore throat, breathlessness or flu-like symptoms). We will phone you, or send a text message, to remind you to take the nose swabs once a week. If you struggle to take the nose swabs yourself, simply let us know and we can arrange for the researcher to visit you and help take the swabs.

We will let you know when a courier will collect the swabs from you – this will happen on a weekly basis. The swabs will be taken to a laboratory team who will do COVID-19 tests on them. This takes up to three days, and we will phone you, or send a text message, with the results.

After the first 12 weeks, we do not need swabs every week. However, we would still like you to collect nose swabs at any point in the study if you think you have fallen ill with fever, cough, sore throat, or breathlessness. At this point, the processing of swabs may change depending on local NHS testing policy

The researcher will visit and collect up to 60ml blood samples after you have been in the study for 12 weeks (3 months), then again at 6 months, 1 year, 2 years and 3 years after you started the study. For the participants unable to donate blood, the researcher will take a finger prick test and blood spot collection. At these visits, you will also complete a questionnaire.

If a nose swab has a positive test result: *We will provide support in terms of your healthcare (either via the phone, or face-to-face), and we will also visit you a few days after your positive result, and then 2 weeks after that. We will inform your GP of your positive result. Public Health England maybe informed in line with their current guidelines. When we visit, we will ask you more questions, and take some more samples, both from the person in the household who has tested positive, and from the rest of the household as well. The samples we will take are: another nose swab, a swab for saliva, a stool sample, and further blood samples (up to 60ml). We may also provide you with a special device for rapid diagnosis to take a nose/throat swab, but this is only if it is approved for use and part of NHS guidelines. If another member of the household tests positive, then we will take additional blood, saliva, nose and throat samples off them as well. The maximum number of times we take samples would be about 4 in a month.*

If, at any point, you become ill or suspect you have COVID-19, you can call the telephone number given on the first page of this information sheet. If you suspect you have COVID-19 and need to talk to someone outside of usual office hours, you should call **NHS ###.**

The study is planned to last for three years. At the moment the study is going to be started for the first 12 weeks (3 months); continuing the study after that will depend on the study team obtaining more funding. However, our intention is to run the study for three years, so we are asking for permission for this up front from you, so that we do not need to keep coming back to you.

We will let you know when the study ends.

What will I have to do if I take part?

If you agree to take part, you will be asked to sign a consent form. You will be provided with a copy of the consent form and the information sheet to keep.

Once you have signed the consent form, you will be asked to follow the study plan (see study timeline diagram below).

You will have to:

- Provide swab of the throat and nose at the beginning, repeated weekly for the first 12 weeks and at any time point you have fallen ill with fever, cough, sore throat, or breathlessness taken by yourself
- Be ready at pre-set timepoints for the courier to collect the swabs (once a week)
- Provide blood samples (or finger prick test, if unable to donate blood)
- Provide saliva swabs (only if you test positive for COVID-19)
- Provide stool sample at week 6-8 and 12-14 (optional), and if you test positive for COVID-19)
- Complete questionnaires (either by yourself or over the telephone)
- Provide information on your health and wellbeing

For peer review only

For peer review only - http://bmjopen.bmj.com/site/about/guidelines.xhtml

Initial contact

Joining COVID-LIV

- Potential participants are contacted for the study team to give a brief explanation about the study, answer any queries and acquire verbal consent.
- A visit by a team of research nurse or doctor is arranged.

Week 0

Initial visit

- The study team will answer any questions and acquire written informed consent.
- Detailed questionnaire will be filled
- Participants will provide blood sample (or fingerprick), nose and throat swabs, nasal strip and saliva.
- The study team will explain how to take self-administered nose swabs and provide with swab kits for the next weeks

Weeks 1 - 11

Weekly questionnaires and swabs

- Participant will fill a brief questionnaire
- Participants will take weekly nose swab
- Participant will provide an optional stool samples at week 6-8.
- Courier will come and collect the samples every week.

Week 12

Questionnaire, blood sample and swabs

- You will complete a detailed questionnaire.
- If you choose to, you can also provide a stool sample at week 12-14.
- We will take a blood sample (or fingerprick), and a nose swab.
- We will phone/text you with the results in 2/3 days.

If you test positive (study nurse visits you)

If you test positive for COVID-19 (this visit will occur a few days later, and again about 2 weeks after that)

We will inform your GP of the positive result.
We will ask some questions about your health.
We will take a blood sample, nose swab, throat swab, nasal strip, a swab for saliva and a stool sample from all members of the household where someone has tested positive. If another person tests positive more samples will be taken off the household, up to 4 in a month.
Also at 3, 6, 12, 24 and 36 months.
We may also take a nose/throat swab using a special device, but this is only if it is approved for us to use.

If additional funding is received, and the study continues

If the study continues past 3 months, this visit will occur 6 months, 1 year, 2 years and 3 years after you consented to join the study

You will complete a detailed questionnaire.
We will take a blood sample.

12 weeks, or up to 3 years, after you join the study

End of your participation

No further visits/samples/data collection will occur once you have reached this point, unless additional funding is received.

For peer review only

What are the benefits and risks of taking part?

The main benefit is that you will know if you have had COVID-19 or not. If you have symptoms, and the test is negative, we will tell you this. This means that, once you have **self-isolated for 7 days** (as per government advice at the beginning of June, if this changes we will let you know), you could still go out to work or to the shops because we will know for sure that you have not got COVID-19. We will also be doing antibody tests on your blood, so you will be tested for immunity as well. However, at the moment we are not sure if these tests means that you will actually be protected from repeat infections in the future. Studying that is one of the aims of the study.

The risks are minor bruising from the blood samples taken, and researchers entering your house. However, the researchers will be wearing Personal Protective Equipment (PPE) at all times, to prevent transfer of COVID-19.

What happens if I change my mind?

If at any point you decide to stop taking part in the study, you can let a member of the research team know. In order for us to understand why participants withdraw from the study, we may ask you why you have decided to withdraw. However, you do not have to give a reason, if do not want to.

If you do decide to stop taking part, this will not affect your current and future medical care, and your legal rights will not be affected in any way.

Information on how we will handle your information in the event of you withdrawing is detailed in Part 2 of this Information Sheet.

What happens when the study stops?

It is intended that the results of the study will be presented at conferences and published in medical journals so that we can explain to the medical community what our research results have shown. Confidentiality will be ensured at all times and you will not be identified in any publication.

Any information derived directly or indirectly from this research, as well as any patents, diagnostic tests, drugs, or biological products developed directly or indirectly as a result of this research may be used for commercial purposes. You have no right to this property or to any share of the profits that may be earned directly or indirectly as a result of this research. However, in signing this form and donating swabs and blood samples for this research, you do not give up any rights that you would otherwise have as a participant in research.

What if there is a problem?

If, at any point, you become ill or suspect you have COVID-19, you can call the telephone number given on the first page of this information sheet. If you suspect you have COVID-19 and need to talk to someone outside of usual office hours, you should call **NHS ###**.

Any complaint about the way you have been dealt with during the study or any possible harm you might suffer will be addressed. Detailed information is given in Part 2 of this information sheet.

Will my taking part in the study be kept confidential?

Yes. All the confidential information about your participation in this study will be kept confidential. Detailed information on this is given in Part 2.

For peer review only

For peer review only - http://bmjopen.bmj.com/site/about/guidelines.xhtml

PART 2: Detailed information about the conduct of the study

Who is running the study?

University of Liverpool is the Sponsor of this study and is responsible for managing it. They are based in United Kingdom. They have asked that the day to day running of the study is carried out by a team based at the Liverpool Clinical Trials Centre (LCTC, part of the University of Liverpool), and the samples you provide will be managed by members of a laboratory team at the University of Liverpool and Liverpool School of Tropical Medicine (LSTM).

The study has been reviewed by the National Research Ethics Service Committee to make sure that the study is scientifically and ethically acceptable.

This study is funded by the National Institute for Health Research (NIHR) Health Protection Research Unit in Emerging and Zoonotic Infections (HPRU-EZI), Centre of Excellence in Infectious Diseases Research (CEIDR) at University of Liverpool in partnership with Public Health England (PHE), Alder Hey Charity and in collaboration with Liverpool School of Tropical Medicine The views expressed are those of the author(s) and not necessarily those of the NIHR or the Department of Health and Social Care.

We will not receive any payment for including you in this study.

How will my information be collected and handled?

University of Liverpool is the Data Controller for this study and will need to use information from this research project.

This information will include your name, initials, date of birth, contact details, postcode and your NHS number (we will request this from your GP). People will use this information to do the research or to check your records to make sure that the research is being done properly.

Individuals from University of Liverpool, the LCTC, LSTM, and relevant regulatory organisations may look at your research records to check the accuracy of the research study.

People who do not need to know who you are will not be able to see your name or contact details. Your data will have a code number instead.

Data will be sent from the University of Liverpool researchers and LSTM to the LCTC. Data may also be sent from your GP to University of Liverpool researcher, who will then send this to LCTC.

We may notify your GP that you are taking part in the study, and if you test positive for COVID-19, for their information.

We will keep all information about you safe and secure.

Once we have finished the study, we will keep the data for a minimum of 10 years, so we can check the results. We will write our reports in a way that no-one can work out that you took part in the study.

What are my choices about how my information is used?

You can stop being part of the study at any time, without giving a reason, but we will keep information about you that we already have.

We need to manage your records in specific ways for the research to be reliable. This means that we won't be able to let you see or change the data we hold about you.

Information sharing for other research

When you agree to take part in a research study, the information about your health and care may be beneficial to researchers running other research studies in this organisation and in other organisations. These organisations may be universities, NHS organisations or companies involved in health and care research in this country or abroad. Your information will only be used by organisations and researchers to conduct research in accordance with the UK Policy Framework for Health and Social Care Research, or equivalent standards.

If you agree to take part in this study, you will have the option to take part in future research using your data saved from this study.

For peer review only

For peer review only - http://bmjopen.bmj.com/site/about/guidelines.xhtml

Where can I find out more about how my information is used?

You can find out more about how we use your information:

- at the LCTC website: www.lctc.org.uk/privacy
- at LSTM website: https://www.lstmed.ac.uk/lstm-privacy-statement
- at www.hra.nhs.uk/information-about-patients
- in the Health Research Authority leaflet available from www.hra.nhs.uk/patientdataandresearch
- by contacting the University of Liverpool Data Protection Officer on LegalServices@liverpool.ac.uk
- by asking one of the research team
- by sending an email to COVID-LIV-FWCom@liverpool.ac.uk, or
- by ringing us on ###

What will happen to the nose and throat swabs, and the blood, saliva and stool samples I give?

Samples collected for use in COVID-LIV

We will use your samples to test for your body's response to the COVID-19 virus. This will be able to tell us whether you have had the infection with no symptoms, in some cases. We will test your blood to see if you have been exposed before to other coronaviruses.

Your samples will be sent to laboratories at the University of Liverpool and LSTM for analysis. These samples will be coded and the researchers carrying out tests on the samples will not be given information they do not need to carry out the tests and analyse the results. Coded is not the same as anonymous. The study ream will need to know who the sample came from to inform you of the results. It will be possible to use the codes to identify that a result is from your sample. However, once we have given you your results, we do not plan to do this unless there is a good reason to do so. We will maintain this information so that we can properly manage the samples. For instance, sometimes we may need to update our record of your clinical details to help us interpret the results of tests.

If you or a member of your household tested positive for COVID-19: We will use your samples for much more detailed and complex tests on the body's response to the COVID-19 virus. We will study the body's response for everyone in the household – this will help understand how people become infected and how some people may resist the virus. We may also test your blood with other viruses as controls, such as the glandular fever virus, flu, and some other common cold viruses. We will look in detail at the responses of specialised white blood cells in your blood called lymphocytes.

We may store some of your blood, or the cells from your blood for up to 25 years after this research has finished. We may also use some of your cells to make what are called "cell lines" – these are cells that can be kept alive in the lab for a long time (maybe forever) and are used to make it easier to detect and study the lymphocyte responses we are interested in.

We will extract DNA to look at your genes by sequencing the whole of your genome. This will help us understand whether certain genes are related to your ability to fight off the COVID-19 coronavirus. This will include some of the genes unique to your individual immune system, called HLA. These are the same tests that are done before organ transplants, and they are used to tailor our research to each person. There will be left over DNA after we have done this, which would be stored, like the cells from your blood, for up to 25 years after the research has finished.

Samples collected for Future Research

We would like your permission to do other research on these stored samples in the future. This would include looking at factors which are involved in fighting off coronaviruses, and other controls for our experiments such as herpes viruses (like glandular fever), flu, and enteroviruses (e.g. common cold viruses), and other common human viruses. We cannot say now all the experiments we might do because new things might be discovered in the future that we would like to investigate.

If you agree that we can store your samples for future research, coded samples will be stored at the University of Liverpool and LSTM. These researchers work closely with other scientists in the UK and elsewhere. We would like to allow other researchers, including those

For peer review only

For peer review only - http://bmjopen.bmj.com/site/about/guidelines.xhtml

who also have ideas about coronaviruses, to apply to use your samples for similar work in the future too.

We would like your permission to allow your samples to be transferred outside of the University of Liverpool and LSTM for purposes including those of coronavirus immunity testing. We are asking this now so we don't have to ask you again in the future.

Any future experiments not related to coronaviruses or other common human virus infections would be approved by a research ethics committee. If you don't want your samples stored, that's fine, you can still take part in the rest of the study, we just won't keep your samples at the end. Or if you are happy with having your samples stored, but not sent to another lab, you can choose this as well.

The samples will be kept in a secure place until we need them; nobody outside of the study will have access to **any** confidential information that you give to us. Confidential details (such as your name, address and GP details) will be kept locally and not made available to collaborators.

What if there is a problem?

If you have a concern about any aspect of this study, you should ask to speak with one of the research team who will do their best to answer your questions.

Every care will be taken in the course of this research study. However, in the unlikely event that you are harmed by taking part in this research project of the study Sponsor (University of Liverpool), the Sponsor holds Insurance for the conduct of clinical research. Compensation may be available and you may have to pay your related legal costs.

Thank you for taking the time to read and consider this information sheet. Should you decide to take part in the study, you will be given a copy of the information sheet and a signed consent form to keep.

For peer review only - http://bmjopen.bmj.com/site/about/guidelines.xhtml

COVID-LIV Study

UNIVERSITY OF LIVERPOOL

Liverpool Household COVID-19 Cohort Study (COVID-LIV)

FOR SITE USE ONLY:

Site Name:			
Household ID:		Participant ID	
Participant Initials:		Participant DOB:	/ /
Participant Postcode:	-		

Adult Consent Form

To be completed by the participant:

Once you have read and understood each statement please enter your initials in each box.	Initial
1. I have read and understood the information sheet for this study. I have had the opportunity to ask questions and have had these answered satisfactorily.	
2. I understand that participation is voluntary and that I am free to withdraw from the study at any time, without giving a reason, and without my care or legal rights being affected. However, the study team may need to collect some limited information for safety reasons.	
3. I give permission for a copy of this fully completed consent form to be sent to the LCTC (where it will be kept in a secure location) to allow confirmation that my consent was given.	
4. I understand that relevant sections of my medical notes and any data collected during the study may be looked at by authorised individuals from the central study team and representatives of the Sponsor, and relevant regulatory authorities. I give permission for these individuals to have access to my records and data.	
5. I understand that my data will be kept by the University of Liverpool and all others archiving data in a confidential manner for up to a maximum of 25 years from the end of the study.	
6. I consent to samples of my blood, saliva and stool, and for nose and throat swabs to be taken and used for this study.	
7. I agree to take part in the above study.	
The statements below are optional (you can still take part in the study even if you do not wish to agree to these):	
8. I agree for data previously collected for other research to be looked at by the research team.	
9. I consent to providing an optional stool sample at week 6-8 and 12-14 for the purpose of this study.	
10. I consent to my blood and stool samples being stored by the University of Liverpool for testing for SARS-CoV-2 immunity, and immunity to other coronaviruses.	
11. I agree for samples collected for future research to be stored at The University of Liverpool and LSTM.	
12. I agree for samples collected for future research to be transferred outside of the University of Liverpool and LSTM, along with a copy of this Consent Form, for different tests for coronavirus immunity.	
13. I agree to allow information or results arising from this study to be used in future healthcare and/or medical research providing my confidentiality is maintained.	
14. I agree to my GP being informed of my participation in the study, and if I test positive for COVID-19.	
15. I agree for my GP to be contacted for information relating to my health records, including the provision of my NHS number.	

For peer review only

For peer review only - http://bmjopen.bmj.com/site/about/guidelines.xhtml

COVID-LIV Study

UNIVERSITY OF LIVERPOOL

Liverpool Household COVID-19 Cohort Study (COVID-LIV)

FOR SITE USE ONLY:

Site Name:			
Household ID:		Participant ID	
Participant Initials:		Participant DOB:	/ /
Participant Postcode:	-		

Adult Consent Form

16. I agree that I may be contacted in the future in relation to this or other related studies.
If you agree to statement 15 provide your details below:

Telephone number:	
Email address:	

To be completed by the participant:

Your full name (please print):			
Your signature:		Date:	

To be completed by the Researcher (after participant has completed the form):

Researcher full name (please print):			
Researcher signature:		Date:	

Please file the original wet-ink copy in the COVID-LIV Investigator Site File, and make two copies: one for the participant and one to be sent to the LCTC.

For peer review only - http://bmjopen.bmj.com/site/about/guidelines.xhtml
